# Comparison of C-reactive protein with distinct hyperinflammatory biomarkers in association with COVID-19 severity, mortality and SARS-CoV-2 variants

**DOI:** 10.3389/fimmu.2023.1213246

**Published:** 2023-06-14

**Authors:** Tudorita Gabriela Paranga, Mariana Pavel-Tanasa, Daniela Constantinescu, Claudia Elena Plesca, Cristina Petrovici, Ionela-Larisa Miftode, Mihaela Moscalu, Petru Cianga, Egidia Gabriela Miftode

**Affiliations:** ^1^ Department of Infectious Diseases (Internal Medicine II), Faculty of Medicine, Grigore T. Popa University of Medicine and Pharmacy, Iasi, Romania; ^2^ St. Parascheva Clinical Hospital for Infectious Diseases, Iasi, Romania; ^3^ Department of Immunology, Faculty of Medicine, Grigore T. Popa University of Medicine and Pharmacy, Iasi, Romania; ^4^ Laboratory of Immunology, St. Spiridon County Clinical Emergency Hospital, Iasi, Romania; ^5^ Department of Preventive Medicine and Interdisciplinarity, Faculty of Medicine, Grigore T. Popa University of Medicine and Pharmacy, Iasi, Romania

**Keywords:** COVID-19, CRP, suPAR, s-TREM-1, HGF, biomarkers, disease severity, mortality

## Abstract

C-reactive protein (CRP) has been one of the most investigated inflammatory-biomarkers during the ongoing COVID-19 pandemics caused by severe acute respiratory syndrome coronavirus-2 (SARS-CoV-2). The severe outcome among patients with SARS-CoV-2 infection is closely related to the cytokine storm and the hyperinflammation responsible for the acute respiratory distress syndrome and multiple organ failure. It still remains a challenge to determine which of the hyperinflammatory biomarkers and cytokines are the best predictors for disease severity and mortality in COVID-19 patients. Therefore, we evaluated and compared the outcome prediction efficiencies between CRP, the recently reported inflammatory modulators (suPAR, sTREM-1, HGF), and the classical biomarkers (MCP-1, IL-1β, IL-6, NLR, PLR, ESR, ferritin, fibrinogen, and LDH) in patients confirmed with SARS-CoV-2 infection at hospital admission. Notably, patients with severe disease had higher serum levels of CRP, suPAR, sTREM-1, HGF and classical biomarkers compared to the mild and moderate cases. Our data also identified CRP, among all investigated analytes, to best discriminate between severe and non-severe forms of disease, while LDH, sTREM-1 and HGF proved to be excellent mortality predictors in COVID-19 patients. Importantly, suPAR emerged as a key molecule in characterizing the Delta variant infections.

## 1 Introduction

The end of 2019 marked the beginning of a difficult period for humanity, the COVID-19 Pandemic, which put pressure on the health system all around the world. A new coronavirus, SARS-CoV-2 (severe acute respiratory syndrome coronavirus-2), first reported in Wuhan, Hubei, China, has widely spread, so that on January 30, 2020, the WHO declared a public health emergency of international interest, and a few weeks later (March 11, 2020) a global pandemic (1, 2).

In order to cope with the large numbers of COVID-19 patients during the peak periods of the pandemic waves, optimizing the hospital resources, by early discharging the patients at no risk of developing a severe form of disease, became a general necessity (1, 2). Since the pandemics’ beginning, it has been widely accepted that the acute respiratory distress syndrome (ARDS) was associated with the severe/critical forms of disease and represented the main cause of death. However, the general clinical features of SARS-CoV-2 infection were heterogeneous and non-specific, covering a large spectrum of respiratory, digestive, cardiovascular, renal, neurological or psychiatric clinical manifestations, and showing unpredictable evolution towards critical illness (respiratory failure, septic shock, and/or multiple organ dysfunction) even in some patients with initial mild or moderate symptoms (3, 4). In this context, only considering the clinical evaluation as a decisive criterion for the early but safe discharge of patients was not possible, and hence, the need and the general effort for establishing laboratory-derived biomarkers that would facilitate the identification of patients at risk for disease progression or fatal outcome (4, 5).

For the confirmed cases of SARS-CoV-2 infection, a series of routinely-investigated biomarkers, including C-reactive protein (CRP), leukocyte count, lactate dehydrogenase (LDH) and alanine transaminase (ALT), was associated with severe disease and mortality (5, 6). Importantly, the high serum levels of CRP, which are normally lacking in viral infections, but observed in the severe cases since the beginning of COVID-19 pandemic, may be explained by the high production of IL-6 accompanying the Macrophage Activation Syndrome (5–8). Consequently, COVID-19 patients developing ARDS have increased plasma levels of pro-inflammatory cytokines, such as interleukins-1 beta and -6 (IL-1β, IL-6), tumor necrosis factor alpha (TNF-α), chemokines—CXCL10 (IP-10), CCL2 (monocyte chemoattractant protein-1, MCP-1), and CCL3 (macrophage inflammatory protein-1 alpha, MIP-1α) (9, 10). These cytokines cause distinct positive feedback loops on other immune cells, contributing to recruiting them, generating an exponential growth of inflammation which ultimately leads to multiple organ damage (9–12). For instance, CCL2, despite being an important player in the antiviral defense, due to its overwhelming secretion caused by the SARS-CoV-2 infection, becomes one of the key contributors in generating ARDS and even death in patients with severe COVID-19 (13). Additionally, Sarif J. et al. identified the soluble urokinase plasminogen activator receptor (suPAR) as a key pathogenic circulating molecule linking the systemic hyperinflammation to a hypercoagulable status in severe and critical COVID-19 patients, and thus, suggested using suPAR as a predictor for disease severity and mortality (14). To counteract the hyperinflammation, the immune system starts then releasing pleiotropic molecules with anti-inflammatory properties, such as the hepatocyte growth factor (HGF) and the soluble form of the triggering receptor expressed on myeloid cells (sTREM-1), such as neutrophils, monocytes and macrophages. These molecules, being produced in high amounts, intensify the coagulation abnormalities and the multiple organ failure, and are associated with disease severity and poor clinical outcome in COVID-19 (15–17). Recent systematic evaluation and meta-analysis studies conducted for identifying valuable biomarkers of disease prognosis and treatment responses in COVID-19 patients, also reported the potential usefulness of fibrinogen, D-dimers or neutrophil-lymphocyte ratio (NLR) (6, 18, 19).

Despite having a generous diversity of inflammatory mediators involved in poor outcomes, it still remains a challenge to determine which of these above-mentioned cytokines or chemokines are the best predictors of disease progression and mortality in COVID-19 patients with various forms of disease, ranging from mild, moderate to severe or critical illness (3).

Being well aware of the important contribution that these biomarkers would have on the management of the SARS-CoV-2 infected patient, we set out to evaluate and compare the prediction efficiency of CRP, the best described biomarker during the COVID-19 pandemic, with the more recent reported modulators with pro-inflammatory (such as suPAR, MCP-1, IL-1β, IL-6, NLR, platelet-lymphocyte ratio (PLR), ESR, ferritin, fibrinogen, and LDH) and anti-inflammatory (sTREM-1 and HGF) properties in a group of patients confirmed with SARS-CoV-2 infection and stratified as mild, moderate and severe cases. Importantly, the cases were investigated between October 2021 and May 2022, time frame which comprised the transition period from Delta to Omicron variants (the end of 2021), and this allowed us to additionally report our biomarkers’ observations to distinct SARS-Co-V-2 variant infections. Here we identified that CRP was the best predictor of disease severity among our investigated biomarkers, with suPAR levels being correlated with Delta infection, while LDH, sTREM-1 and HGF proved to best discriminate between survivors and non-survivors.

## 2 Materials and methods

### 2.1 Study participants and serum collection

Blood samples were collected at *St. Parascheva* Clinical Hospital for Infectious Diseases (Iasi, Romania) between October 2021 and May 2022 from SARS-CoV-2 infected individuals at hospital admission. Patients’ inclusion criteria were: (1) adult patients of either sex; (2) SARS-CoV-2 infection confirmed by qRT-PCR tests through nasopharyngeal and oropharyngeal swab samples; (3) need for hospitalization; (4) either status of vaccination anti-SARS-CoV-2: yes or no; (5) given consent for the recruitment into the study. Exclusion criteria were as follows: (1) age < 18 years; (2) administration of anti-inflammatory medication prior hospital admission; (3) immunocompromised patients (e.g. HIV/AIDS, transplant, cancer), (4) pregnancy; (5) no inclusion in other clinical studies.

The patients were next stratified according to disease severity in mild, moderate and severe cases, based on the signs and symptoms at hospital admission, and according to the international clinical spectrum guidelines of SARS-CoV-2 infection. More precisely, the mild group comprised the cases with few symptoms (low fever, cough, myalgias, fatigue, anorexia) without evidence of viral pneumonia or hypoxia. The moderate group comprised the cases with clinical signs of pneumonia (fever, cough, dyspnea, fast breathing), but no signs of severe pneumonia, including SpO_2_ ≥ 90% on room air. In the severe group, we categorized the patients with clinical signs of pneumonia (fever, cough, dyspnea) plus one of the following: respiratory rate > 30 beaths/min; severe respiratory distress; or SpO_2_ < 90% on room air. The patients who were discharged from the hospital were designated as survivors, while those who died during hospitalization were named non-survivors or deceased. Since the first two cases of Omicron infection were officially reported in Romania on the 4^th^ of December 2021 by the Romanian National Institute of Public Health (RNIPH), all the cases before 1^st^ of December 2021 were categorized as Delta SARS-CoV-2 infections, and the cases hospitalized in December were excluded from our study. As from the 1st week of 2022, the Omicron variant of concern represented more than 60% of the nasopharyngeal/oropharyngeal swab samples sequenced by RNIPH (20, 21), the cases after 1^st^ of January 2022 were categorized as predominantly Omicron infections.

Inclusion in the study did not influence the patients’ management and the therapeutic decision was left at the discretion of the attending physicians. This study has been reviewed and approved by the institutional ethics committees (*St. Parascheva* Clinical Hospital for Infectious Diseases, Iasi), and an informed consent was obtained from all the participants in this study. More precisely, 153 participants agreed for CRP and other pro-inflammatory biomarkers’ testing (fibrinogen, ferritin, LDH, NLR – neutrophil-lymphocyte ratio, PLR – platelet-lymphocyte ratio), of which 140 subjects also agreed for inflammatory cytokine profile (suPAR, sTREM-1, MCP-1, HGF, IL-1β, IL-6). The information related to age and gender was included in a database together with a unique identifier, in order to keep the sample’s identity unknown to the researcher. Overall patients’ characteristics are detailed in **Supplementary Table 1**.

### 2.2 Sample processing

Blood samples were collected in vacutainers with no anticoagulant and processed within 6 hours of receipt. More precisely, blood was spun at 2000 G for 5 min, and the serum was separated, while aliquots of 500 μl were kept for storage at -80°C until further analysis of the cytokines’ profile.

• **for assessing CRP, classical inflammatory biomarkers and hematological parameters**

The analysis of CRP, ferritin, complete blood count, coagulation profile (fibrinogen, D-Dimers, prothrombin index), multiple organ failure biomarkers (aspartate transaminase (AST), ALT, total bilirubin, urea, creatinine, potassium, sodium, ionized calcium, chloride) was performed immediately after serum separation at *St. Parascheva* Hospital Laboratory using designated *in vitro* diagnosis kits for the automated platforms Rx Imola and Cobas. CRP levels were detected using the automated immunoturbidimetric Randox Full-Range Assay on the RxImola platform with a wide measuring range of 1.8-1650 mg/L.

• **for assessing the cytokine profile**

The cytokine profiles were assessed at the Immunology Laboratory of *St Spiridon* County Emergency Hospital, Iasi. After thawing, the samples were centrifuged at 2,000 G for 5 min. The analysis of serum concentrations of various inflammatory cytokines/chemokines (sTREM-1, MCP-1/CCL-2, HGF, IL-1β, IL-6) was performed using a human pre-mixed multi-analyte kit (LXSAHM-05) from R&D systems and performed on a Luminex 100/200 analyzer. A 2-fold dilution was performed for all samples using the calibrator diluent RD6-52 before processing according to the manufacturer’s instructions. Briefly, 50 μl of standards and samples were mixed with 50 μl magnetic microparticle cocktail and left for a 2 hours incubation at room temperature on a horizontal orbital microplate shaker set at 800 rpm. Following a washing procedure comprising 3 washes and the use of a magnetic device designated to accommodate the microplate, 50 μl of diluted biotin-antibody cocktail were added to each well and the plate was incubated for 1 hour at room temperature on the shaker at 800 rpm. After another washing step, 50 μl of the diluted streptavidin-PE solution was added for 30 min at room temperature on the shaker. After a final wash, the microparticles were resuspended in 100 μl of wash buffer, incubated for 2 minutes and then the plate was read within 60 minutes. For suPAR analysis, the suPARnostic AUTO Flex ELISA kit from ViroGates (E001) was used. The samples were used undiluted. Briefly, 15 μl of standards, controls or samples were pre-mixed with 135 μl of conjugation working solution, mix from which 100 μl was further transferred to the ELISA plate and left for 1 hour incubation at room temperature in the dark. The plate was then washed 3 times with 250 μl of wash buffer using the TECAN hydroflex platform. Then, 100 μl of TMB (tetramethylbenzidine) solution was added to each well and the enzymatic reaction was stopped after 20 minutes. The plate absorbance was read at 450 nm with the reference filter of 650 nm using the TECAN reader infinite 200 pro.

### 2.3 Statistical analysis

Statistical analysis was performed using Graph Pad Prism, v5 (Graph Pad Software, San Diego, CA, USA) and SPSS, v25 (IBM SPSS Software, Chicago, IL, USA). Data are presented as scatter dots, bars with information about the mean and SEM, or box and whiskers plots. Each figure legend contains the relevant statistical information: the *n*, total number of participants, the significance *p*-value and the corresponding statistical tests performed. All data were checked for both normality and variance using the Shapiro-Wilk test. The parametric data were analyzed using the unpaired t-test and one-way ANOVA with *Post-hoc* Tukey’s Multiple Comparison test. The majority of the data were non-parametric and the statistical tests applied were: Mann-Whitney test (the non-parametric counterpart to unpaired t-test), and Kruskal-Wallis with Dunn’s Multiple Comparison test (the non-parametric counterpart to one-way ANOVA). Spearman’s correlation coefficients (R) were used to assess positive or negative associations between measured variables. R values between 0.2-0.39 were treated as weak, between 0.4-0.59 as moderate, and between 0.6-0.79 as very strong correlation factors. Each linear regression graph was performed using Graph Pad Prism v5 and shows the best-fit line with the 95% confidence band. The coefficient of determination R-squared (R2) was used as a goodness-of-fit measure and the F-test to determine the level of significance for each linear regression. The receiver operating characteristic (ROC) curves were generated in SPSS, v25 in order to compare the sensitivity (sn) *vs.* specificity (sp) across a range of possible cut-off values, and the area under those curves (AUC) was used as a measure of test performance. The optimal cut-off values were determined as previously described in (22). The Kaplan-Meier survival curves, together with the univariate and multivariate analysis, were also generated using SPSS, v25. The *p* values less than 0.05 was considered statistically significant.

## 3 Results

### 3.1 Serum CRP, suPAR, sTREM-1 and HGF levels increased in severe COVID-19 patients

**Figure 1:**
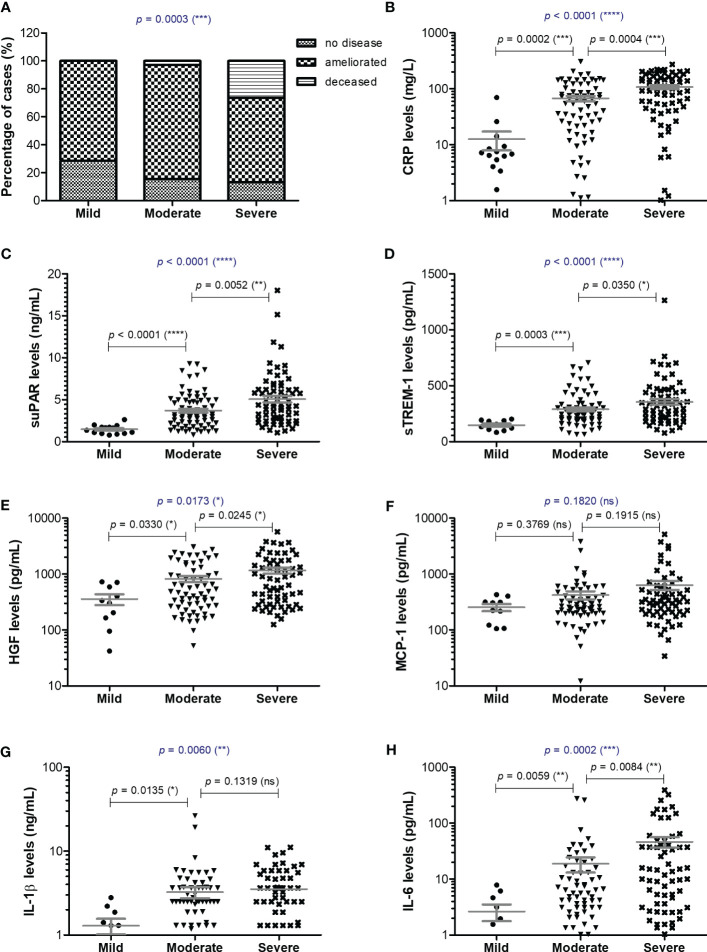
Serum profile of CRP and pro-inflammatory cytokines in mild, moderate and severe COVID-19 disease. **(A)** Patients’ discharge status (no disease, ameliorated symptoms or deceased) for each category of COVID-19 disease: mild, moderate, severe (***p < 0.001; chi-squared test). Serum levels of **(B)** CRP, **(C)** suPAR, **(D)** sTREM-1, **(E)** HGF, **(F)** MCP-1, **(G)** IL-1β, **(H)** IL-6 for each category of COVID-19 disease: mild, moderate or severe. The gray lines represent the mean ± SEM (****p < 0.0001, ***p < 0.001, **p < 0.01, *p < 0.05, ns – not significant; **(A)** chi-squared test; **(B-H)** Kruskal-Wallis with Dunn’s Multiple Comparison test).

Blood samples were collected from 153 SARS-CoV-2 infected patients in the first day of hospitalization, between October 2021 and May 2022. The patients were categorized based on disease severity (patients’ characteristics are displayed in **Supplementary Table 1**) in mild (14 cases [9.15%]), moderate (71 cases [46.41%]), and severe (68 cases [44.44%]) cases. All mild cases survived and were hospitalized for a median of 6 days (IQR 5-9), while the moderate severe patients registered a 2.82% death rate for a median of 10 days of hospitalization (IQR 10-12), which significantly increased among severe COVID-19 individuals, reaching 26.5% mortality rate and a hospitalization period of 13 days (IQR 8-18) (**Figure 1A** and **Supplementary Table 1**). The median age was 55 years (IQR 45.5-66) for mild cases, which increased to 67 (IQR 57-73) and 70 (IQR 57.5-79.5) for moderate and severe patients. Each group was further stratified by gender and the female to male ratios were 1.8, 0.78 and 0.79 for mild, moderate and severe COVID-19 patients, suggesting an increased risk for moderate and/or severe SARS-CoV-2 infection among male individuals (Odd Ratio of 2.3). In the mild group, 11 cases (78.6%) had increased serum CRP levels over the normal range of 0-5 mg/L, while 61 (85.9%) and 65 (95.6%) cases had abnormally increased CRP levels in the moderate and severe groups. More precisely, the serum CRP levels ranged from 12.59 mg/L [95% CI 2.52-22.66] in mild cases to 67.25 mg/L [95% CI 2.52-22.66] and 107.7 mg/L [95% CI 90.61-124.9] for moderate and severe patients, respectively (**Figure 1B** and **Supplementary Table 2**). The differences in CRP levels among the three groups of COVID-19 severity were significant for both female (*p* < 0.0001) and male (*p* = 0.0012) subgroups. These changes were sustained by similar changes in the serum levels of newly characterized biomarkers of systemic inflammation (suPAR, sTREM-1, HGF) and classical biomarkers (cytokines - IL-1β, IL-6, chemokines – MCP-1/CCL2, and hematological/biochemical markers – NLR, PLR, ESR, fibrinogen, ferritin and LDH) (**Table 1**).

**Table 1:** The values of inflammatory biomarkers for each category of COVID-19 patients: mild, moderate and severe.

Analyte	Mild		Moderate		Severe		p-value
	Median [IQR]	Mean [95% CI]	Median [IQR]	Mean [95% CI]	Median [IQR]	Mean [95% CI]	
**CRP (mg/L)**	7.01 [5.05-10.57]	12.59 [2.516-22.66]	54.14 [16.45-102.9]	67.25 [52.69-81.81]	99.18 [45.64-166]	107.7 [90.61-124.9]	**< 0.0001**
**F**	6.45 [4.72-17.69]	15.33 [-1.162-31.83]	29.56 [9.34-85.91]	52.47 [31.75-73.19]	94.95 [40.67-146]	98.2 [74.88-121.5]	**< 0.0001**
**M**	7.2 [4.19-11.32]	7.644 [2.051-13.24]	72.35 [29.42-122.1]	78.71 [58.4-99.01]	113.2 [55.07-184.2]	115.3 [90.07-140.5]	**0.0012**
**suPAR (ng/mL)**	1.51 [0.99-1.78]	1.47 [1.17-1.77]	3.14 [2.05-4.92]	3.68 [3.19-4.18]	4.31 [2.71-6.23]	5.06 [4.29-5.82]	**< 0.0001**
**F**	1.12 [0.98-1.83]	1.36 [1.009-1.713]	2.99 [1.82-4.92]	3.6 [2.79-4.41]	4.3 [2.64-7.08]	5.39 [3.96-6.81]	**< 0.0001**
**M**	1.68 [1.13-2.18]	1.66 [0.87-2.45]	3.31 [2.34-4.97]	3.75 [3.11-4.39]	4.54 [2.73-6.22]	4.8 [3.96-5.64]	**0.0030**
**sTREM-1 (pg/mL)**	144.5 [106.4-189.9]	146.8 [115.7-178]	249.9 [194.8-341.9]	288.8 [252.5-325]	318.3 [211.3-453.7]	354.8 [304.3-405.3]	**< 0.0001**
**F**	139 [94.46-188.1]	140.7 [90.88-190.5]	240.9 [155.8-303.9]	250.1 [197.4-302.7]	317.4 [189.7-448.9]	353.5 [259.5-447.5]	**0.0051**
**M**	160.7 [115-192.4]	156 [89.65-222.4]	275 [198-428.8]	315.6 [266.1-365]	319.2 [212.5-466]	355.7 [297.2-414.2]	**0.0188**
**HGF (pg/mL)**	318.7 [147.6-619.3]	357.1 [180.4-533.8]	478.6 [262.7-1184]	818.5 [628.7-1008]	705.4 [295.8-1691]	1157 [872.3-1441]	**0.0173**
**F**	184.5 [82.13-399.1]	238.6 [29.81-447.4]	366.5 [205.3-1021]	662.7 [410.2-915.2]	891.6 [322.5-1733]	1086 [733.4-1439]	**0.0042**
**M**	555.5 [326.2-723]	534.9 [190.8-879.1]	598.9 [265.9-1587]	926.3 [652.9-1200]	569.1 [272.3-1629]	1208 [774.8-1642]	0.3299
**MCP-1 (pg/mL)**	265.1 [118.1-337.8]	254.2 [169.6-338.7]	265.1 [199.6-429.1]	421.2 [283.9-558.5]	323.5 [210.3-551]	634.6 [404.2-865]	0.1820
**F**	220.5 [118.1-337.8]	231.5 [112.2-350.9]	248.7 [179-328.9]	266 [213.9-318]	341.6 [232.3-607.7]	805.2 [340.3-1270]	**0.0136**
**M**	309.8 [156.1-398.2]	288.1 [75.83-500.3]	285.1 [203.7-596.2]	528.7 [301.1-756.2]	301.4 [179.3-544]	510.1 [285.5-734.7]	0.8543
**IL-1β (pg/mL)**	1.301 [0.743-1.958]	1.291 [0.6735-1.909]	2.493 [1.301-3.656]	3.238 [2.268-4.208]	3.251 [1.301-4.754]	3.533 [2.891-4.174]	**0.0060**
**F**	1.126 [0.0915-2.356]	1.229 [0.05828-2.4]	2.493 [0.954-3.638]	3.068 [1.133-5.004]	2.493 [1.874-4.754]	3.226 [2.395-4.056]	**0.0312**
**M**	1.356 [1.041-1.758]	1.385 [0.7805-1.989]	2.493 [1.301-3.709]	3.356 [2.32-4.391]	3.638 [1.301-5.578]	3.757 [2.8-4.713]	0.2042
**IL-6 (pg/mL)**	1.75 [0.4-5.02]	2.64 [0.6754-4.605]	5.65 [2.59-13.85]	18.78 [7.371-30.19]	12.77 [3.235-40.95]	46.26 [26.52-65.99]	**0.0002**
**F**	0.5 [0.0775-1.95]	0.9683 [-0.292-2.228]	4.23 [2.33-10.57]	8.161 [4.032-12.29]	15.85 [4.64-49.07]	61.47 [21.3-101.6]	**< 0.0001**
**M**	5.4 [2.623-7.42]	5.148 [1.169-9.126]	5.65 [2.59-24.4]	26.13 [7.025-45.24]	9.69 [2.565-39.32]	35.15 [16.36-53.95]	0.4600
**NLR**	2.675 [1.33-4.795]	3.73 [1.922-5.538]	5.076 [3.226-7.288]	6.239 [5.059-7.419]	9.254 [3.421-13.72]	10.31 [8.442-12.17]	**< 0.0001**
**F**	3.864 [2.013-8.795]	5.096 [0.6332-9.559]	5.281 [3.06-8.084]	6.975 [5.001-8.948]	8.612 [3.317-12.85]	9.412 [7.248-11.58]	**0.0001**
**M**	2.469 [1.124-3.247]	2.972 [0.8438-5.099]	4.933 [3.383-6.814]	5.29 [4.335-6.245]	9.87 [4.745-15.64]	11.44 [8.111-14.77]	0.1033
**PLR**	150.8 [116.8-208.8]	199.7 [112.6-286.9]	221.3 [145.9-336.4]	277.8 [232.5-323.1]	263.9 [178.8-415.9]	327.7 [270.3-385.1]	**0.0177**
**F**	144.1 [121.4-192.1]	205.1 [67.48-342.8]	233.3 [184.6-304.3]	283.2 [221.8-344.5]	256.9 [176.3-422.5]	354.2 [241.4-467]	**0.0346**
**M**	189.1 [99.83-280.6]	190 [67.33-312.7]	213 [127.1-336.8]	273.6 [206.3-340.9]	275 [178.8-421]	306.8 [250.6-363]	0.1966
**ESR (mm/h)**	29.5 [16.5-53.75]	38.5 [20.6-56.4]	60.5 [40-80]	64.31 [55.97-72.66]	70 [42.5-102]	71.86 [62.53-81.2]	**0.0055**
**F**	39 [20-75]	45.11 [19.62-70.6]	60 [30-75]	62.22 [47.95-76.49]	60 [43.75-107]	71.63 [57.54-85.72]	0.1041
**M**	15 [6.5-52.5]	26.6 [-5.064-58.26]	61 [46-85]	65.84 [55.26-76.42]	75 [36-100]	72.06 [58.95-85.17]	**0.0254**
**Fibrinogen (g/L)**	3.475 [3.215-4.08]	3.592 [3.248-3.936]	4.78 [3.74-5.37]	4.681 [4.404-4.958]	5.37 [4.29-5.82]	5.269 [4.907-5.63]	**< 0.0001**
**F**	3.61 [3.19-4.42]	3.721 [3.197-4.245]	4.61 [3.61-5.37]	4.611 [4.158-5.065]	4.875 [3.785-5.618]	4.821 [4.403-5.24]	**0.0202**
**M**	3.44 [3.055-3.625]	3.36 [2.934-3.786]	4.875 [4.14-5.498]	4.736 [4.375-5.097]	5.82 [4.545-7.11]	5.632 [5.082-6.181]	**0.0007**
**Ferritin (μg/L)**	213.6 [100.8-490.1]	408.4 [25.4-791.3]	498.2 [209.9-1261]	1034 [515.6-1553]	862.5 [353.3-1794]	1568 [779.1-2357]	**0.0029**
**F**	102.2 [76.43-253.8]	152.5 [-23.47-328.5]	280.7 [103.7-468.8]	378.2 [200-556.3]	658.1 [298.5-1674]	1133 [711.9-1553]	**0.0003**
**M**	454.5 [213.6-1220]	664.2 [-179-1507]	916.2 [457.3-1633]	1510 [645.1-2374]	920.6 [362.7-2000]	1891 [530.6-3252]	0.3693
**LDH (U/L)**	168.5 [133.3-196.8]	166 [143.1-188.9]	280 [202.3-386]	294.3 [264.1-324.5]	338 [240-481]	396.4 [339.9-452.8]	**< 0.0001**
**F**	162 [132-211]	161.3 [121.5-201]	225.5 [184-298.8]	250.7 [218.6-282.7]	382 [308-531.5]	420.9 [350.6-491.2]	**< 0.0001**
**M**	177 [148.5-194.5]	172.6 [139.4-205.8]	304.5 [248.3-413.8]	325.8 [280.7-370.8]	311 [211.3-472.5]	377.6 [291.5-463.8]	**0.0210**

CRP, C-reactive protein; suPAR, soluble urokinase plasminogen activator receptor; sTREM-1, soluble triggering receptor expressed on myeloid cells-1; HGF, hepatocyte growth factor; MCP-1, monocyte chemoattractant protein-1; IL-1β, interleukin-1 beta; IL-6, interleukin-6; NLR, neutrophil-lymphocyte ratio; PLR, platelet-lymphocyte ratio; ESR, erythrocyte sedimentation rate; LDH, lactate dehydrogenase; F, females; M, males; IQR, interquartile range; CI, confidence interval; p, statistical significance coefficient. The bold values are the statistically significant p-values.

For instance, the serum suPAR levels showed 2.51- and 3.45-fold increases in moderate and severe cases, respectively, when compared to the mild SARS-CoV-2 infected patients (1.47 ng/mL [95% CI 1.17-1.77], *p* < 0.0001) with no significant differences among the female and male subgroups (**Figure 1C**). Interestingly, also the serum levels of soluble molecules with anti-inflammatory function, such as the soluble form of triggering receptor expressed on myeloid cells 1 (sTREM-1) and hepatocyte growth factor (HGF), showed similar significant increasing trends (*p* < 0.0001, and *p* = 0.0173, respectively). While sTREM-1 serum levels showed 1.97- and 2.42- fold increases in moderate and severe cases irrespective of gender (**Figure 1D**), the HGF levels largely depended on gender with 2.8- and 4.6-fold increases in moderate and severe female cases and only 1.7- and 2.3- fold increases in moderate and severe male patients when compared to the mild subjects’ values (238.6 pg/mL [95% CI 29.81-447.4] for females, and larger values of 534.9 pg/mL [95% CI 190.8-879.1] for males; **Figure 1E** and **Table 1**). The MCP-1/CCL2 values also described an increasing trend from mild to severe cases, but with no significant differences between the three groups (*p* = 0.1820, **Figure 1F**). On the other hand, IL-1β showed a similar 2.6-fold change irrespective of gender in the moderate and severe cases (*p* = 0.006) when compared to the mild group characterized by a mean value of 1.29 pg/mL [95% CI 0.67-1.91] (**Figure 1G**). IL-6 values, similar to HGF, showed significant increases among the three severity groups only for females (p < 0.0001), but with more prominent differences as the total levels varied from 2.64 pg/mL [95% CI 0.68-4.61] in mild cases to 18.78 pg/mL [95% CI 7.37-30.19] in moderate, and 46.26 pg/mL [95% CI 26.52-65.99] in severe COVID-19 patients (**Figure 1H**).

**Figure 2:**
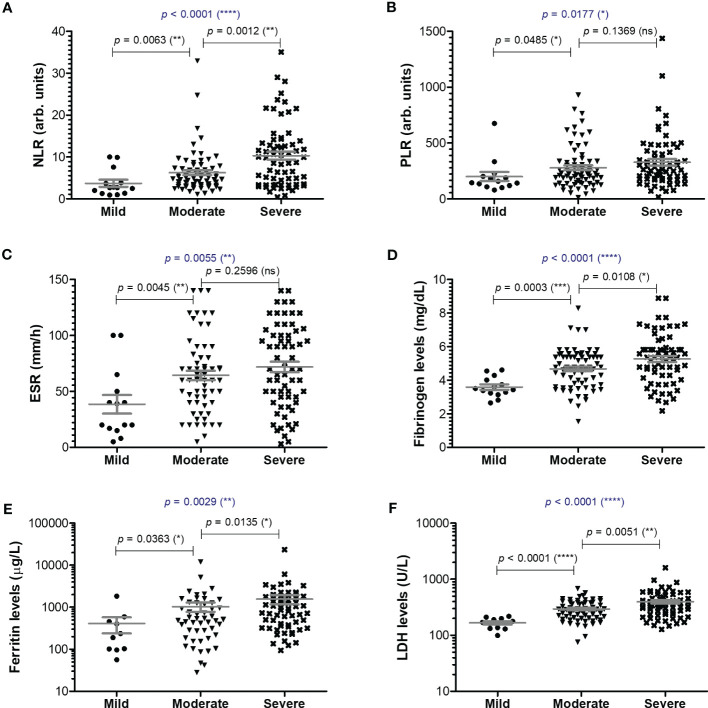
Serum profile of common pro-inflammatory biomarkers in mild, moderate and severe COVID-19 disease. Serum levels of **(A)** NLR (neutrophil-lymphocyte ratio), **(B)** PLR (platelet-lymphocyte ratio), **(C)** ESR (erythrocyte sedimentation rate), **(D)** fibrinogen, **(E)** ferritin, **(F)** LDH for each category of COVID-19 disease: mild, moderate or severe. The gray lines represent the mean ± SEM (****p < 0.0001, ***p < 0.001, **p < 0.01, *p < 0.05, ns – not significant; Kruskal-Wallis with Dunn’s Multiple Comparison test).

Among the classical hematological and biochemical inflammatory biomarkers, neutrophil-lymphocyte ratio (NLR), platelet-lymphocyte ratio (PLR), erythrocyte sedimentation rate (ESR), fibrinogen, ferritin, and lactate dehydrogenase (LDH) showed a general similar significant rising trend as the CRP levels from mild to moderate and then, to severe subjects (**Figure 2**). However, the described differences for NLR (*p* = 0.0001), PLR (*p* = 0.0346) and ferritin serum levels (*p* = 0.0003) were significant only for the subgroup of female patients (**Table 1**). The pro-inflammatory landscape noticed in the severe form of COVID-19 was hence associated with hematological changes, including the described increase of NLR and PLR values. The WBC had a general tendency to increase from mild to severe, and most importantly, both the total number and percentage of neutrophils and eosinophils increased at the expense of lymphocytes which significantly decreased from 1.29 x 10^3^/μl (95% CI 0.94—1.64) in mild cases to 0.79 x 10^3^/μl (95% CI 0.58-0.96) in severe patients. The platelet counts did not significantly differ among the three groups of subjects. The present data clearly show that CRP and the other investigated pro-inflammatory biomarkers gradually increase from mild to moderate and/or severe COVID-19 cases and this is supported by an expansion of neutrophils and eosinophils together with a reduction in the lymphocyte count.

### 3.2 CRP levels are not influenced by age in COVID-19 patients

**Figure 3:**
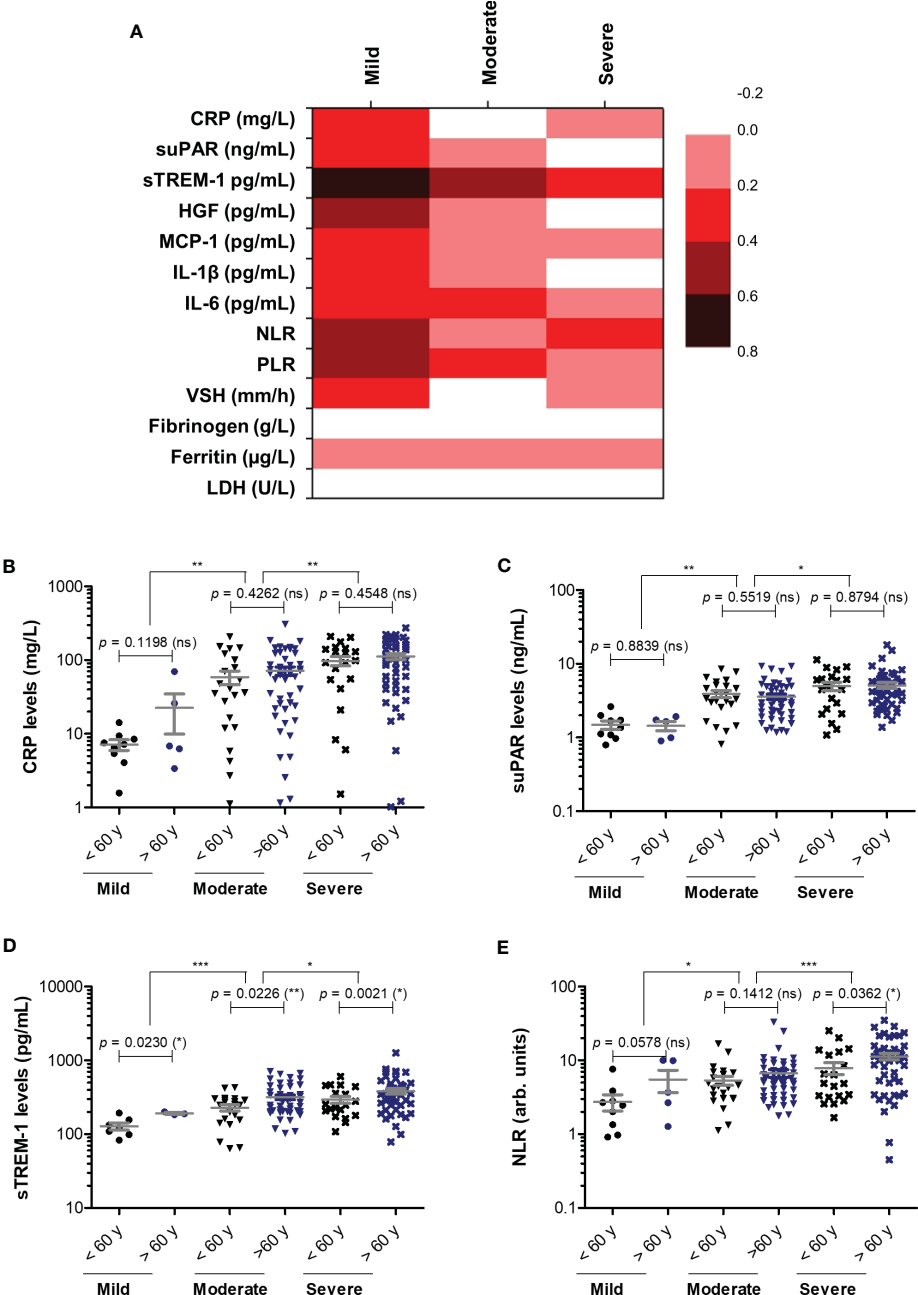
Correlation of pro-inflammatory biomarkers with age in mild, moderate and severe SARS-CoV-2 infections. **(A)** Heat map of correlation coefficients (R) for each category of COVID-19 disease: mild, moderate, severe (0.2-0.39: weak; 0.4-0.59: moderate; 0.6-0.8 strong). **(B)** CRP levels, **(C)** suPAR levels, **(D)** sTREM-1 levels, and **(E)** NLR values in mild, moderate and severe COVID-19 patients stratified by age (younger or older than 60 years). The gray lines represent the mean ± SEM (***p < 0.001, **p < 0.01, *p < 0.05, ns – not significant; Kruskal-Wallis with Dunn’s Multiple Comparison test).

Next, we wondered whether the serum levels of CRP and of the other investigated inflammatory biomarkers vary according to age (**Figure 3A**). First, CRP and suPAR levels did not seem to be influenced by age in our study group (**Figures 3B, C**). Interestingly, significant correlations (total correlation R = 0.35, *p* < 0.0001) with patients’ age were noticed for sTREM-1 values for all three categories of mild (R = 0.68), moderate (R = 0.45) and severe (R = 0.21) cases. Indeed, when stratified by age, the subgroup of subjects older than 60 years constantly showed higher values of sTREM-1 (an average of 1.4-fold increase) in each subgroup: mild, moderate or severe (**Figure 3D**). Significant general correlations were also observed for NLR (*p* = 0.0016) and PLR (*p* = 0.0036) with a moderate value of 0.45 within the group of mild subjects (**Figure 3A**). A moderate variation with age was also observed in the mild group for HGF (R = 0.51, **Figure 3E**). Based on these observations, we can conclude that CRP and the majority of inflammatory biomarkers (except sTREM-1, NLR and PLR) had a weak variation by age in COVID-19 patients.

### 3.3 sTREM-1, HGF, ESR, fibrinogen and LDH best correlated with CRP in severe COVID-19 patients

**Figure 4:**
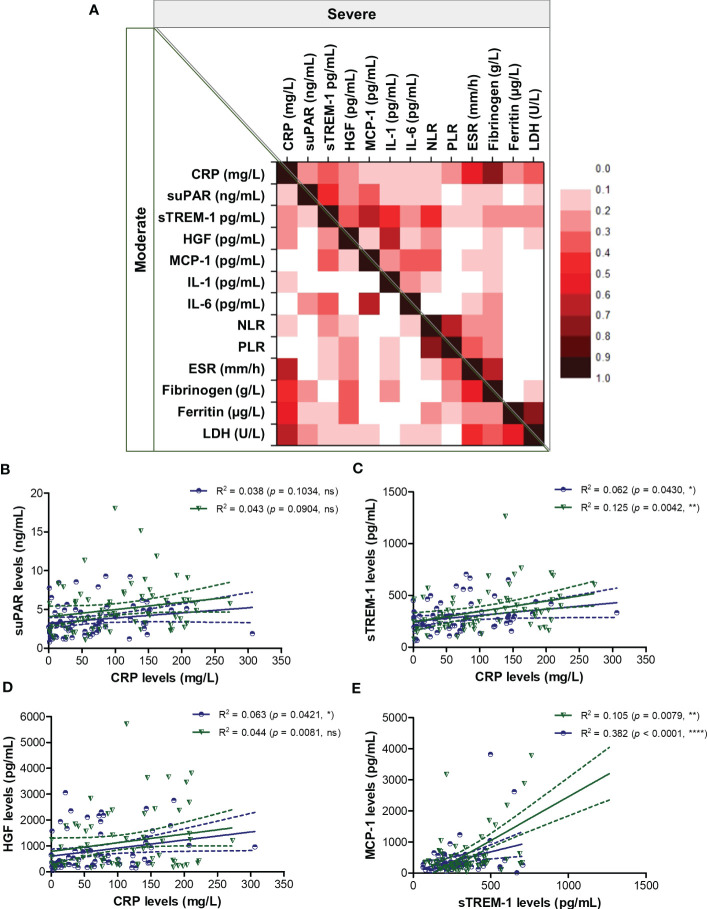
Regression statistics describing the association between CRP and other pro-inflammatory biomarkers in moderate and severe cases of SARS-CoV-2 infection. **(A)** Heat map of correlation coefficients (R) for each category of COVID-19 disease: moderate (left-bottom corner) and severe (right-upper corner). Linear regression analysis for **(B)** CRP and suPAR levels, **(C)** CRP and sTREM-1 levels, **(D)** CRP and HGF levels, and **(E)** sTREM-1 and MCP-1 levels in, moderate and severe COVID-19 patients (****p < 0.0001, **p < 0.01, *p < 0.05, ns – not significant; Spearman test). The blue lines and dots correspond to moderate cases, while the green lines and dots state for severe cases.

**Figure 5:**
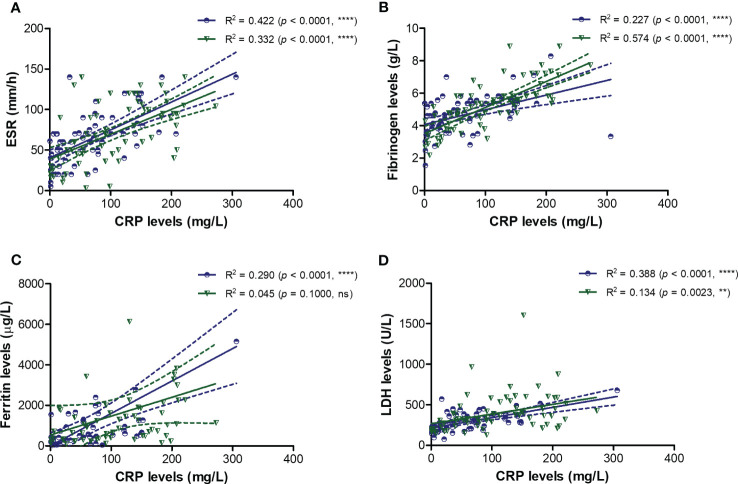
Regression statistics describing the association between CRP and classical pro-inflammatory biomarkers in moderate and severe cases of SARS-CoV-2 infection. Linear regression analysis for CRP and **(A)** ESR, **(B)** fibrinogen, **(C)** ferritin, and **(D)** LDH in moderate and severe COVID-19 patients (****p < 0.0001, ***p < 0.001, **p < 0.01, ns – not significant; Spearman test). The blue lines and dots correspond to moderate cases, while the green lines and dots state for severe cases.

To better picture the general landscape of CRP and of the other 12 biomarkers serum levels in moderate and severe COVID-19 patients, we further investigated the correlation coefficients between any combination of bio-signatures (**Figure 4A**). First, we noticed higher associations among CRP, suPAR, sTREM-1, HGF, MCP-1, IL-1β, IL-6 and NLR in the severe group when compared to moderate subjects. Next, CRP showed significant moderate correlation with sTREM-1 and HGF in both moderate and severe patients, while suPAR showed good correlations with sTREM-1, HGF and MCP-1 only in the severe group (**Figures 4A–D**). Interestingly, sTREM-1 showed moderate association with HGF, MCP-1 and IL-6 in the moderate group, but good and very good correlations with all the above-mentioned biomarkers in severe cases (**Figure 4E**). When investigating the correlations with classical hematological and biochemical inflammatory signatures, we observed a clear association of ESR, fibrinogen, LDH with CRP in both moderate and severe patients (**Figure 5**). These results suggest that the severe form of COVID-19 is characterized by a strong pro-inflammatory phenotype, and most importantly, by a clear association between CRP and sTREM-1, a recently described biomarker of COVID-19 severity and mortality.

### 3.4 SARS-CoV-2-infection with Delta variants, compared to Omicron, induced higher inflammatory responses

**Figure 6:**
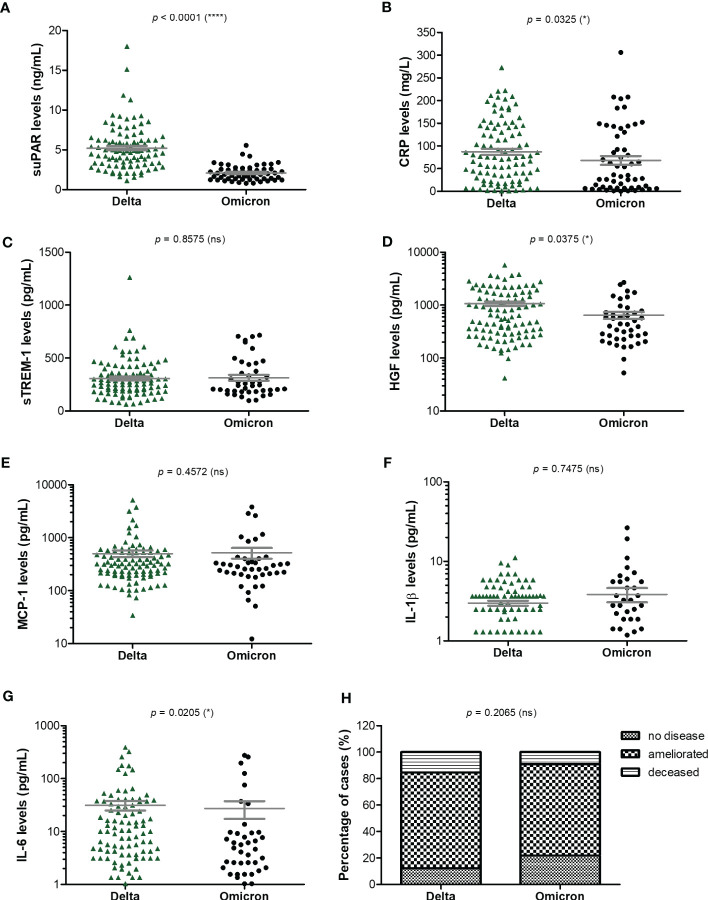
Serum profile of CRP and pro-inflammatory cytokines in Delta and Omicron SARS-CoV-2 infections. Serum levels of **(A)** suPAR, **(B)** CRP, **(C)** sTREM-1, **(D)** HGF, **(E)** MCP-1, **(F)** IL-1β, **(G)** IL-6 for each category of SARS-CoV-2 infection: Delta or Omicron. The gray lines represent the mean ± SEM (****p < 0.0001, *p < 0.05, ns – not significant; two-tailed Mann-Whitney test). **(H)** Patients’ discharge status (no disease, ameliorated symptoms or deceased) for each category of SARS-CoV-2 infection: Delta or Omicron (ns – not significant; chi-squared test).

As the Delta SARS-CoV-2 infection was associated with a higher death rate worldwide when compared to the other variants (23), we next aimed to investigate the differences, if any, in our selected inflammatory biomarkers. Indeed, in our group of investigated patients, the death rate was 15% among the Delta-infected individuals, and lowered to 9% for the Omicron-infection (**Supplementary Figure 1**). Interestingly, the most prominent difference was noticed for the serum suPAR levels, where significantly higher levels were observed for the Delta variant infections in both moderate and severe cases (moderate cases: 4.61 ng/mL for Delta *vs.* 1.9 ng/mL for Omicron; severe cases: 5.74 ng/mL for Delta *vs*. 2.37 ng/mL for Omicron), **Figure 6A** and **Table 2**. Importantly, CRP and most of the other biomarkers showed overall higher values for the Delta infection compared to the other variants (**Figures 6B–G**). Among those, HGF, IL-1β and fibrinogen showed significant differences in the group of moderate COVID-19 cases. For instance, HGF levels reached 580.8 pg/mL [95% CI 327.5-1610] in the Delta moderate infection compared to 299.2 pg/mL [95% CI 195.7-625.1] in the Omicron infection. For fibrinogen, increased levels were noticed in 87% of the Delta infected individuals compared to only 48% for the Omicron infections. A similar trend was also observed for ESR and LDH (**Supplementary Figure 2**). Most of these described differences were significant in the case of moderate disease severity. Interestingly, these differences were attenuated in the severe COVID-19 cases. In accordance with an overall higher pro-inflammatory response induced by the Delta variant, the percentage of severe cases was also increased (50% of severe cases within the Delta infection group *vs.* 34.5% of severe cases within the Omicron group; *p* = 0.0015; **Figure 6H**).

**Table 2:** The values of inflammatory biomarkers for each category of COVID-19 patients (mild, moderate and severe) based on SARS-CoV-2 variant infection, Delta or Omicron.

Analyte	Mild		Moderate			Severe		
	Delta	Omicron	Delta	Omicron	*p*-value	Delta	Omicron	*p*-value
**CRP (mg/L)**	5.38 [1.57-6.45]	7.54 [6.24-14.18]	56.91 [20.45-107.8]	35.18 [8.32-103.3]	0.3762	99.77 [50.22-164.8]	92.28 [28.25-183.2]	0.7533
**suPAR (ng/mL)**	1.99 [1.12-2.63]	1.36 [0.97-1.72]	4.605 [2.985-5.525]	1.9 [1.295-2.665]	**< 0.0001**	5.74 [3.83-7.11]	2.37 [1.71-3.2]	**< 0.0001**
**sTREM-1 (pg/mL)**	109.1 [82.89-121.7]	183.7 [132.7-193.6]	260.3 [191.3-336.8]	243.2 [199.1-486.6]	0.1125	317.4 [212.4-452.1]	319.2 [207.2-464.4]	0.8121
**HGF (pg/mL)**	204.1 [42.15-301.5]	400.6 [165-710.4]	580.8 [327.5-1610]	299.2 [195.7-625.1]	**0.0322**	827 [303.7-1762]	569.1 [286.9-1489]	0.3542
**MCP-1 (pg/mL)**	106.6 [105.9-315.7]	306.8 [217.5-404]	307.1 [201.8-528.2]	253.5 [184.2-330.9]	0.2062	322.4 [207.7-544]	335.3 [209.7-857.4]	0.8211
**IL-1β (pg/mL)**	1.301 [0-1.301]	1.41 [0.95-2.21]	2.493 [1.301-3.638]	2.104 [0.954-5.541]	**0.0405**	3.638 [1.301-4.754]	3.251 [1.301-5.541]	0.7272
**IL-6 (pg/mL)**	0.1 [0.01-1.95]	3.15 [0.5-6.16]	6.025 [3.11-18.01]	4.5 [2.07-8.813]	0.3049	15.11 [4.89-44.78]	6.16 [1.81-37.04]	0.147
**NLR**	2.469 [0.9167-3.864]	2.678 [1.349-7.59]	5.195 [3.313-7.912]	4.933 [2.742-6.888]	0.4234	8.505 [3.392-13.74]	10.63 [3.566-13.85]	0.6818
**PLR**	144.1 [135-189.1]	157.6 [107.8-227.9]	231.8 [187.5-336.6]	164.7 [109.6-325.8]	0.0955	249 [178.3-404.5]	302.6 [182.5-429.4]	0.5476
**ESR (mm/h)**	20 [5-100]	39 [17-50]	67.5 [47.75-80]	60 [26.25-81]	0.3479	75 [45-105]	55 [32-96.25]	0.4037
**Fibrinogen (g/L)**	4.29 [2.82-4.61]	3.44 [3.24-3.74]	5.065 [4.253-5.58]	3.82 [3.365-5.075]	**0.0088**	5.37 [4.545-5.82]	5.05 [3.473-6.08]	0.3042
**Ferritin (μg/L)**	236.6 [236.6-236.6]	190.5 [99.31-525.6]	525.2 [189-1301]	444.5 [261.6-1019]	0.6605	837 [366.5-1624]	988.8 [316.6-2830]	0.7174
**LDH (U/L)**	137 [130-211]	170 [147-194.5]	295 [214.3-387.8]	225.5 [187.5-317.5]	0.1088	362 [272-525]	331.5 [176-422.3]	0.1471

CRP, C-reactive protein; suPAR, soluble urokinase plasminogen activator receptor; sTREM-1, soluble triggering receptor expressed on myeloid cells-1; HGF, hepatocyte growth factor; MCP-1, monocyte chemoattractant protein-1; IL-1β, interleukin-1 beta; IL-6, interleukin-6; NLR, neutrophil-lymphocyte ratio; PLR, platelet-lymphocyte ratio; ESR, erythrocyte sedimentation rate; LDH, lactate dehydrogenase; p, statistical significance coefficient. The bold values are the statistically significant p-values. The different coloring was performed in order to make it easier to discriminate between the three different groups: mild (gray color), moderate (blue color) and severe (peach color).

### 3.5 CRP and pro-inflammatory cytokines’ levels at hospital admission were higher in the severe COVID-19 patients who did not survive

**Figure 7:**
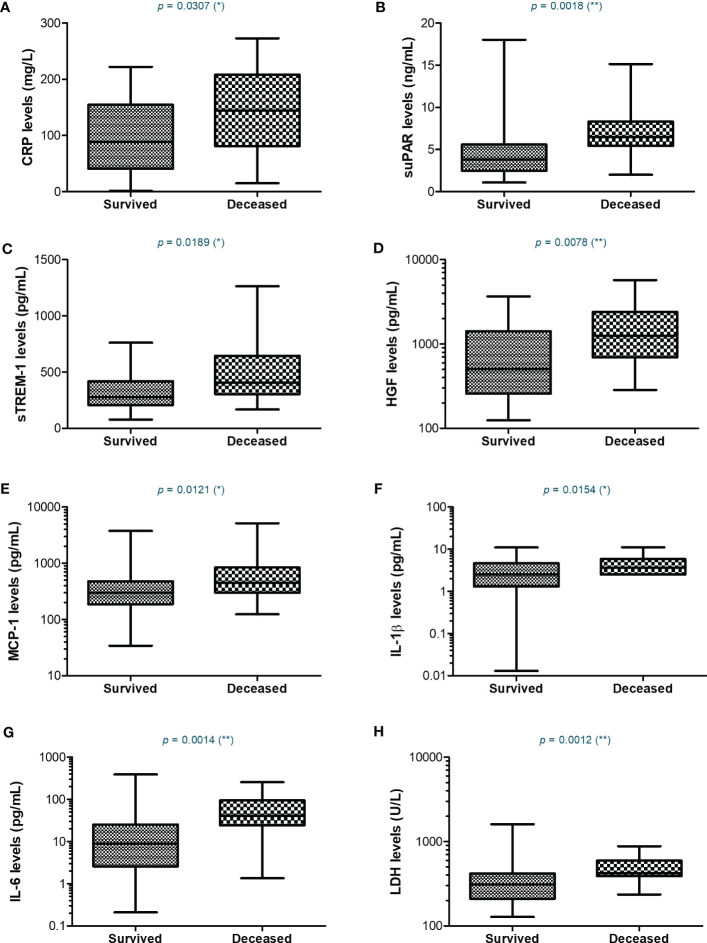
Serum profile of CRP and pro-inflammatory cytokines in severe COVID-19 patients stratified based on survival. Box and whiskers representation of **(A)** CRP, **(B)** suPAR, **(C)** sTREM-1, **(D)** HGF, **(E)** MCP-1, **(F)** IL-1β, **(G)** IL-6, and **(H)** LDH serum levels for each category of SARS-CoV-2 severe infection: survived or deceased (**p < 0.01, *p < 0.05, ns – not significant; two-tailed Mann-Whitney test).

Since most of the deceased patients were from the severe category of COVID-19 patients (26.5% mortality rate), we next wondered which were the discrepancies, if any, between the severe subjects that survived and those who died. For CRP, the mean values were higher with 45% in the deceased group compared to the survivors’ group (**Figure 7**). Similarly, suPAR, sTREM-1, and IL-1β initial levels were higher by 45-50% in the deceased group, while HGF, MCP-1, and IL-6 initial serum levels were even higher, showing an increase of 75-80% compared to the survivors’ group (**Figure 7** and **Supplementary Table 3**). Importantly, among the hematological and biochemical inflammatory biomarkers, only LDH showed a significant increase in the deceased group, but only of 29% (**Supplementary Table 3**). These data reveal that the initial high levels of CRP and cytokines correlated with an increased mortality risk.

As vaccination could have influenced the outcome of patients, we also evaluated the vaccination status among the subjects included in this study. For the mild group, 9 out of 14 cases were vaccinated (64.3%), while the rate of vaccination was significantly lower among moderate and severe cases (22.5% and 17.6%, respectively, **Supplementary Figure 3A**). Among severe cases, no differences in the vaccination rates were observed between survivors and non-survivors (**Supplementary Figure 3B**). Consistently, no positive association between the vaccination status and mortality emerged (**Supplementary Figure 4**), probably also due to the relatively low percentage of general vaccination rate of only 24% in our study group. No consistent significant laboratory differences were noticed between previously vaccinated and non-vaccinated subjects for each category of disease severity (**Supplementary Table 4**).

### 3.6 CRP associated with severe COVID-19, suPAR with Delta variant, while sTREM-1, HGF and LDH with mortality prediction

**Figure 8:**
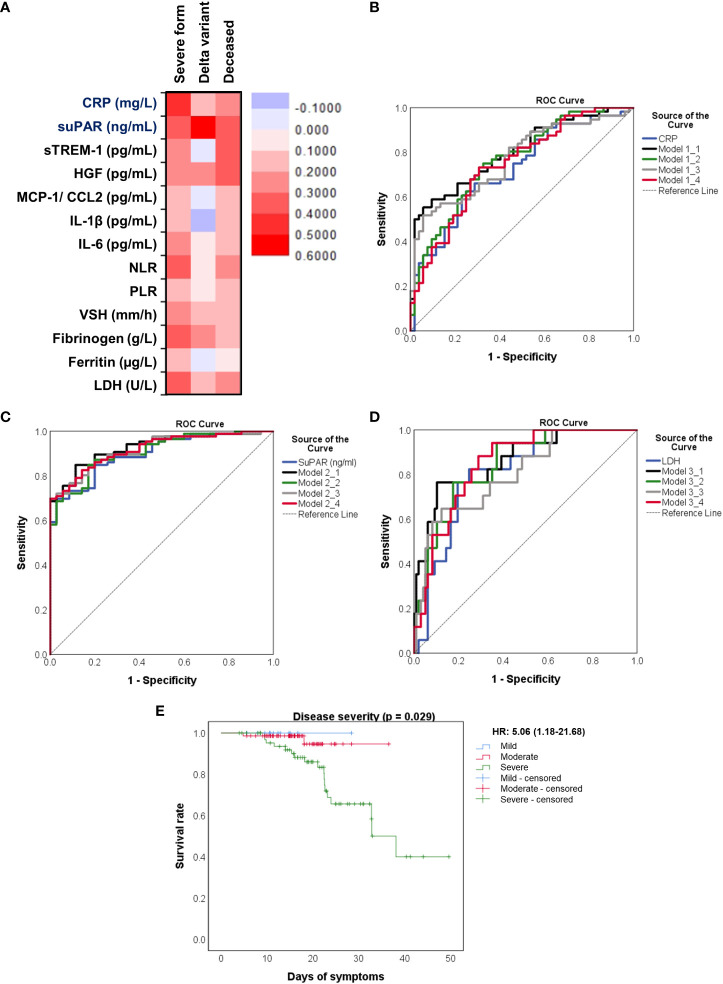
Distinct pro-inflammatory biomarkers associate with disease severity, Delta variant infection or increased death rate. **(A)** Heat map of correlation coefficients of pro-inflammatory biomarkers with disease severity, Delta variant infection or deceased status. ROC curves generated for the **(B)** association of CRP and other pro-inflammatory biomarkers with severe COVID-19, **(C)** association of suPAR and other pro-inflammatory biomarkers with Delta variant infection, **(D)** association of LDH and other inflammatory biomarkers with mortality. The mathematical models from **(B–D)** are detailed in **Supplementary Tables 3–5**, respectively. **(E)** Kaplan-Meier survival curves for each category of COVID-19 patients: mild, moderate and severe.

We next aimed to investigate which of the inflammatory biomarkers were associated with disease severity, SARS-CoV-2 Delta variant and mortality prediction. Among all the markers, the best correlation with the severe form of COVID-19 was observed for the serum CRP levels (R = 0.415, *p* < 0.001, **Figure 8A**), results confirmed also by the ROC analysis (AUC = 0.720, 95% CI 0.625-0.816, *p*< 0.0001). A cut-off value of 76.07 mg/L for CRP yielded a sensitivity of 0.66 and a specificity of 0.71 (**Table 3**). For predicting the severe form of COVID-19, higher AUC values were achieved when CRP was analyzed together with the other inflammatory markers which also showed significant independent associations (Model1_1). The association of CRP with cytokines (suPAR, sTREM-1, HGF, IL-6 – Model 1_2) gave a similar AUC value as the association of CRP with the classical inflammatory biomarkers (NLR, PLR, ESR, fibrinogen, ferritin, LDH – Model 1_3): 0.766 (95% CI 0.678-0.855, *p* < 0.0001) *vs.* 0.769 (95% CI 0.680-0.857, *p* < 0.0001) – see **Figure 8B** and **Supplementary Table 5**. Importantly, CRP analyzed together with any of the following markers, suPAR, sTREM-1 or HGF reached significantly higher AUC values (0.744, 0.738, and 0.731, respectively) than CRP alone. To validate these observations, we also performed univariate and multivariate analysis for the above established cut-off values in relation to age (older than 60 years), gender and vaccination status. As shown in **Supplementary Table 6**, only CRP and NLR could serve as independent predictors for disease severity.

**Table 3:** Statistical evaluation of pro-inflammatory biomarkers in association with severe COVID-19.

Analyte	AUC	S.E.	*p-*value	95% Confidence Interval	Cut-off value	Sensitivity	Specificity
**CRP (mg/L)**	0.720	0.049	**0.000**	0.625-0.816	76.07	0.661	0.712
**suPAR (ng/mL)**	0.698	0.050	**0.000**	0.599-0.797	3.76	0.643	0.673
**sTREM-1 (pg/mL)**	0.674	0.052	**0.002**	0.572-0.777	247.27	0.696	0.635
**HGF (pg/mL)**	0.613	0.054	**0.042**	0.507-0.719	777.52	0.500	0.731
MCP-1 (pg/mL)	0.590	0.055	0.108	0.483-0.697	267.75	0.625	0.558
IL-1β (pg/mL)	0.582	0.055	0.141	0.474-0.690	3.16	0.464	0.692
**IL-6 (pg/mL)**	0.680	0.051	**0.001**	0.580-0.781	7.31	0.643	0.692
**NLR**	0.692	0.052	**0.001**	0.590-0.793	8.21	0.571	0.827
**PLR**	0.634	0.054	**0.016**	0.527-0.741	242.99	0.625	0.712
**ESR (mm/h)**	0.633	0.054	**0.017**	0.527-0.738	73.50	0.518	0.731
**Fibrinogen (g/L)**	0.703	0.050	**0.000**	0.606-0.801	4.64	0.732	0.558
**Ferritin (µg/L)**	0.658	0.053	**0.005**	0.555-0.761	620.80	0.643	0.635

CRP, C-reactive protein; suPAR, soluble urokinase plasminogen activator receptor; sTREM-1, soluble triggering receptor expressed on myeloid cells-1; HGF, hepatocyte growth factor; MCP-1, monocyte chemoattractant protein-1; IL-1β, interleukin-1 beta; IL-6, interleukin-6; NLR, neutrophil-lymphocyte ratio; PLR, platelet-lymphocyte ratio; ESR, erythrocyte sedimentation rate; LDH, lactate dehydrogenase; AUC, area under curve; p, statistical significance coefficient. The bold values are the statistically significant p-values.

When examining the inflammation caused by the Delta variant, suPAR levels showed the highest correlation (R = 0.548, *p* < 0.0001) and an outstanding AUC value of 0.912 (955 CI 0.860-0.963, *p* < 0.0001). For suPAR, the cut-off value of 3.24 ng/mL yielded a sensitivity of 0.77 and a specificity of 0.91 (**Table 4**). All the other markers showed moderate or low differentiation capacity between Delta and Omicron infections (**Figure 8C** and **Supplementary Table 7**). As shown in **Supplementary Table 8**, the multivariate analysis also confirmed that suPAR could indeed serve as an independent discriminator between the two variant infections (Delta *vs.* Omicron).

**Table 4:** Statistical evaluation of pro-inflammatory biomarkers in association with Delta variant infection.

Analyte	AUC	S.E.	*p-*value	95% Confidence Interval	Cut-off value	Sensitivity	Specificity
CRP (mg/L)	0.577	0.062	0.199	0.455-0.698	65.36	0.616	0.571
**suPAR (ng/mL)**	0.912	0.026	**0.000**	0.860-0.963	3.24	0.767	0.914
sTREM-1 (pg/mL)	0.560	0.060	0.312	0.442-0.679	246.06	0.616	0.571
**HGF (pg/mL)**	0.659	0.055	**0.007**	0.553-0.766	434.61	0.630	0.600
MCP-1 (pg/mL)	0.580	0.059	0.177	0.465-0.695	316.67	0.493	0.686
IL-1β (pg/mL)	0.534	0.063	0.564	0.411-0.658	2.27	0.603	0.543
**IL-6 (pg/mL)**	0.670	0.057	**0.004**	0.559-0.781	9.87	0.534	0.829
NLR	0.570	0.061	0.239	0.451-0.689	6.34	0.548	0.600
PLR	0.584	0.064	0.161	0.458-0.710	202.78	0.671	0.486
**ESR (mm/h)**	0.627	0.058	**0.033**	0.513-0.742	67.50	0.548	0.657
**Fibrinogen (g/L)**	0.635	0.063	**0.023**	0.512-0.759	3.90	0.849	0.543
Ferritin (µg/L)	0.521	0.062	0.725	0.399-0.643	466.15	0.644	0.457
**LDH (U/L)**	0.655	0.060	**0.009**	0.538-0.773	254.00	0.753	0.600

CRP, C-reactive protein; suPAR, soluble urokinase plasminogen activator receptor; sTREM-1, soluble triggering receptor expressed on myeloid cells-1; HGF, hepatocyte growth factor; MCP-1, monocyte chemoattractant protein-1; IL-1β, interleukin-1 beta; IL-6, interleukin-6; NLR, neutrophil-lymphocyte ratio; PLR, platelet-lymphocyte ratio; ESR, erythrocyte sedimentation rate; LDH, lactate dehydrogenase; AUC, area under curve; p, statistical significance coefficient. The bold values are the statistically significant p-values.

The best predictors for mortality were represented by the initial values of LDH, HGF, sTREM-1, followed by suPAR and IL-1β serum levels. This observation resulted from computing both the correlations and AUC values for those biomarkers, and the identified cut-off values were: 380 U/L for LDH, 5.2 ng/mL for suPAR, 803 pg/mL for HGF, 334 pg/mL for sTREM-1, and 3.44 pg/mL for IL-1β (**Table 5**). Importantly, the association of LDH with the other biomarkers yielded an improved AUC value of 0.851 (95% CI 0.769-0.933, *p* < 0.0001) when compared to LDH alone (AUC 0.809, 95% CI 0.710-0.907, *p* < 0.0001), see **Figure 8D** and **Supplementary Table 9**. The Kaplan-Meier survival curves confirmed these observations, as the severe COVID-19 patients (**Figure 8E**) and those with values higher than the indicated cut-off scores had a poorer prognosis than those with mild/moderate disease or lower values (**Supplementary Figures 5, 6**). Furthermore, we performed univariate and multivariate Cox regression analysis, and identified that LDH could serve as a promising independent prognostic factor for patients with COVID-19, as shown in **Table 6**.

**Table 5:** Statistical evaluation of pro-inflammatory biomarkers in association with mortality.

Analyte	AUC	S.E.	*p-*value	95% Confidence Interval	Cut-off value	Sensitivity	Specificity
**CRP (mg/dL)**	0.698	0.069	**0.010**	0.562-0.834	79.12	0.765	0.604
**suPAR (ng/mL)**	0.771	0.060	**0.000**	0.653-0.889	5.20	0.765	0.780
**sTREM-1 (pg/mL)**	0.767	0.066	**0.001**	0.637-0.896	334.06	0.765	0.747
**HGF (pg/mL)**	0.797	0.054	**0.000**	0.692-0.902	802.76	0.824	0.703
**MCP-1 (pg/mL)**	0.701	0.067	**0.009**	0.570-0.832	389.32	0.588	0.758
**IL-1β (pg/mL)**	0.785	0.048	**0.000**	0.691-0.879	3.44	0.706	0.692
**IL-6 (pg/mL)**	0.752	0.062	**0.001**	0.631-0.873	14.86	0.765	0.736
**NLR**	0.685	0.069	**0.016**	0.550-0.820	8.39	0.588	0.670
PLR	0.524	0.074	0.758	0.378-0.669	242.99	0.529	0.549
ESR (mm/h)	0.562	0.067	0.418	0.431-0.693	73.50	0.529	0.626
Fibrinogen (g/L)	0.594	0.066	0.220	0.464-0.724	5.07	0.588	0.560
**Ferritin (µg/L)**	0.703	0.058	**0.008**	0.590-0.816	1017.50	0.706	0.681
**LDH (U/L)**	0.809	0.050	**0.000**	0.710-0.907	379.50	0.824	0.758

CRP, C-reactive protein; suPAR, soluble urokinase plasminogen activator receptor; sTREM-1, soluble triggering receptor expressed on myeloid cells-1; HGF, hepatocyte growth factor; MCP-1, monocyte chemoattractant protein-1; IL-1β, interleukin-1 beta; IL-6, interleukin-6; NLR, neutrophil-lymphocyte ratio; PLR, platelet-lymphocyte ratio; ESR, erythrocyte sedimentation rate; LDH, lactate dehydrogenase; AUC, area under curve; p, statistical significance coefficient. The bold values are the statistically significant p-values.

**Table 6:** Univariate and multivariate Cox regression analysis of paraclinical and clinical variables in COVID-19 patients.

Variable	Univariate analysis			Multivariate analysis		
	HR	95% CI	*p*-value	HR	95% CI	*p*-value
CRP (mg/L)	2.364	0.844-6.623	0.102			
suPAR (mg/mL)	7.747	2.807-21.384	**< 0.0001**	6.688	1.692-26.435	**0.007**
sTREM-1 (pg/mL)	5.109	1.798-14.522	**0.002**	1.901	0.513-7.044	0.336
HGF (pg/mL)	4.121	1.570-10.819	**0.004**	1.334	0.309-5.762	0.699
MCP-1 (pg/mL)	2.917	1.143-7.444	**0.025**	0.323	0.084-1.239	0.099
IL-1 (pg/mL)	3.251	1.289-8.203	**0.013**	0.814	0.225-2.940	0.753
IL-6 (pg/mL)	5.276	1.903-14.628	**0.001**	10.358	2.238-47.943	**0.003**
NLR	2.120	0.746-6.026	0.159			
Ferritin (μg/L)	4.257	1.693-10.707	**0.002**	0.809	0.262-2.495	0.713
LDH (U/L)	5.740	1.897-17.373	**0.002**	6.794	1.945-23.733	**0.003**
Severe (yes)	5.367	1.206-23.892	**0.027**	1.408	0.273-7.259	0.683
Age (> 60 years)	1.141	0.378-3.442	0.815			
Gender (M)	1.359	0.537-3.436	0.518			
Vaccination (no)	1.830	0.641-5.223	0.259			

CRP, C-reactive protein; suPAR, soluble urokinase plasminogen activator receptor; sTREM-1, soluble triggering receptor expressed on myeloid cells-1; HGF, hepatocyte growth factor; MCP-1, monocyte chemoattractant protein-1; IL-1β, interleukin-1 beta; IL-6, interleukin-6; NLR, neutrophil-lymphocyte ratio; LDH, lactate dehydrogenase; HR, hazard ratio; CI, confidence interval; p, statistical significance coefficient. The bold values are the statistically significant p-values.

At this point we were able to conclude that the initial values of distinct inflammatory biomarkers are good or excellent predictors for disease severity (e.g., CRP), Delta variant infection (e.g., suPAR) and mortality (e.g., LDH, sTREM-1, HGF, suPAR).

### 3.7 Disease severity and mortality associated a higher rate of comorbidities: thrombocytopenia, diseases of the circulatory system and liver

**Figure 9:**
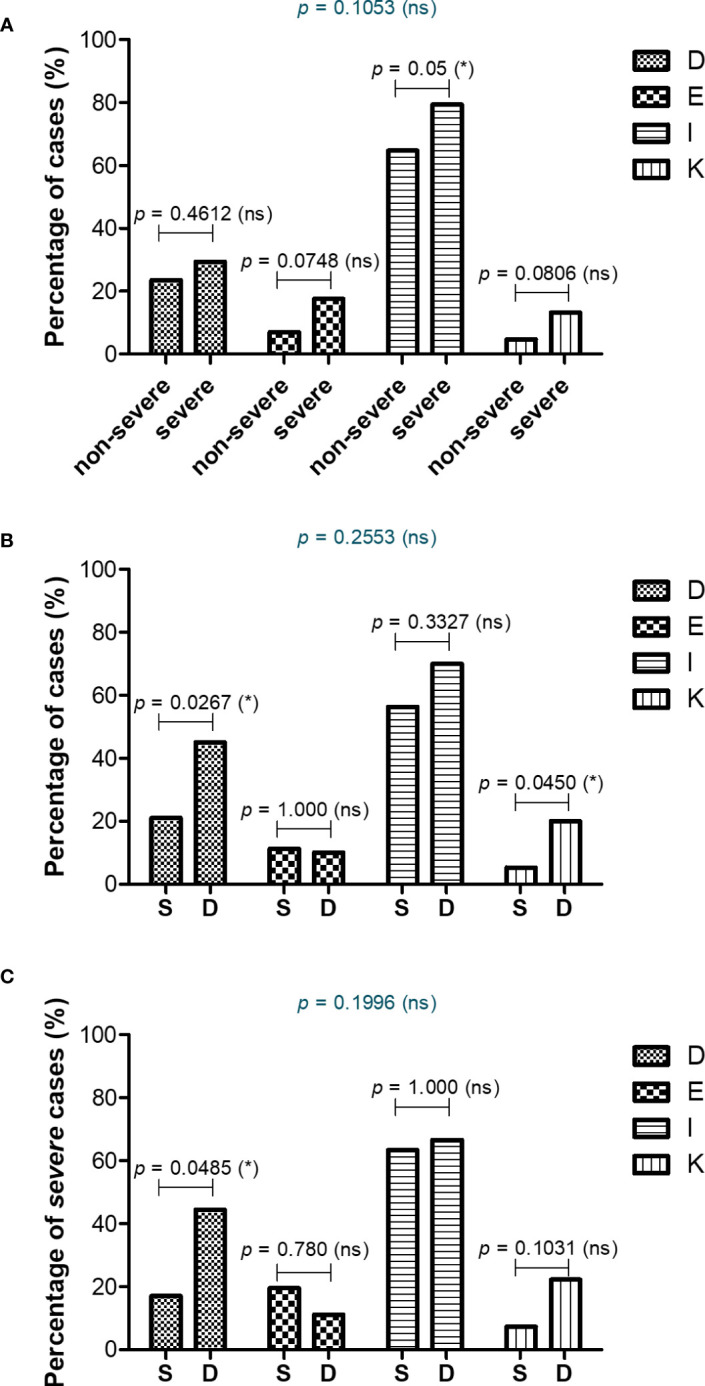
Comorbidities associated with COVID-19 severity and mortality. Differences in comorbidity rates **(A)** between non-severe and severe COVID-19 patients, **(B)** between survived and deceased COVID-19 patients, and **(C)** between survived and deceased severe COVID-19 cases (*p < 0.05, ns – not significant; chi-squared test). D = Diseases of the blood involving the immune mechanism (anemia, purpura and other hemorrhagic conditions), E = Endocrine and metabolic disorders, I = Diseases of the circulatory system, K = Diseases of liver and gallbladder.

As the panel of our inflammatory biomarkers did not yield outstanding associations with disease severity and mortality, we hypothesized that additional criteria might have influenced the COVID-19 disease evolution. Thus, we next investigated the comorbidities’ rate among severe and non-severe patients or between those that survived or died. As expected, more comorbidities were present in severe subjects, and among those, the endocrine and metabolic disorders, together with liver diseases showed an increasing trend, while the diseases of circulatory system yielded a significant rise from 65% in non-severe cases to 80% among severe subjects (**Figure 9A** and **Supplementary Figure 7A, B**). When comparing the survivors’ group with the deceased one, we noticed significant changes among the rate of thrombocytopenia and other blood diseases (from 21% in the survivors’ group to 45% in the deceased group) and liver diseases (showing an increase from 5% to 21% in deceased subjects, who showed either cirrhosis, fatty liver or hepatic failure – **Figures 9B, C** and **Supplementary Figures 7C, D**). Next, we investigated the mortality prediction given by CRP and inflammatory cytokines (those biomarkers that yielded significant differences among survivors and deceased individuals in the severe group). Among those, suPAR and IL-6 generated the highest AUC values of 0.748 and 0.763, respectively. A combined model of all inflammatory biomarkers reached a similar AUC of 0.757 (Model 4_1), while when combined with the presence of comorbidities (Model 4_2) the prediction model reached an excellent AUC of 0.824 (**Supplementary Table 10**). These data are valuable as they point out the importance of comorbidities in influencing the COVID-19 evolution in severe patients.

As the fatal outcome could also be influenced by the therapeutic management, we next investigated the effect exerted by the anti-inflammatory therapy on the survival rate among COVID-19 severe cases. Thus, we categorized the severe cases based on the therapeutic strategies applied by physicians: 18 patients received Tocilizumab, an anti-IL-6 monoclonal antibody, in the first hours-days of hospital admission (median 0 days [IQR 0-1]), 30 patients received Anakinra, an IL-1R antagonist, at a median of 2 days after hospital admission, and the rest 20 severe cases were not administered any of the above anti-interleukin therapies (thus, being designated as the non-AIT group) – **Supplementary Tables 11, 12**. The anti-interleukin therapy did not prove significant beneficial effects on the overall survival rate, with Anakinra treatment apparently associating a higher death rate of 43% (**Supplementary Figure 8A** and **Supplementary Table 11**). Interestingly, the group of severe cases receiving Tocilizumab had higher levels of ferritin and LDH at hospital admission than the non-AIT group, and comprised 4 non-survivors out of 18 subjects. On the contrary, the group of cases receiving Anakinra had higher levels of multiple inflammatory biomarkers compared to the non-AIT group, including CRP (median value of 144.9 mg/L), suPAR (median value of 5.98 ng/mL), sTREM-1 (median value of 380.7 pg/mL), fibrinogen, ferritin and LDH (**Supplementary Figures 8, 9** and **Supplementary Table 12**). Importantly, no statistical differences in co-administered medications or co-morbidities were observed among the three analyzed groups of severe cases (**Supplementary Table 12**).

### 3.8 Discussions

Multiple studies reporting the immunological, hematological, biochemical and coagulation perturbations caused by COVID-19 have emerged during the past 2 years, delineating the hyperinflammatory state that culminates with the cytokine storm and the multiple organ system failure, which are associated with a high risk of mortality [systematically reviewed by Qin et al. (6)]. Interestingly, many of these studies have centered their investigations on certain parameters and tackled their potential associations with disease severity, mortality or comorbidities (24, 25). For instance, separate studies, collected in a recently published systematic review and meta-analysis, have identified a general pro-inflammatory hypercytokinemia profile (IL-1β, IL-6, IL-8, IL-10, IL-18, TNF-α, IFN-y, MCP-1), augmented by high serum levels of CRP and ferritin, hematological abnormalities (dominated by lymphocytopenia and neutrophilia), coagulation dysfunction (indicated by increased D-dimer, fibrinogen and abnormal APTT and PT) and multiple organ injury (revealed by increased LDH, creatinine, urea, AST, ALT, total bilirubin), to be associated with COVID-19 severity and/or mortality (6). Additional studies, have focused on disparate biomarkers, investigating either the role of suPAR in relation to vascular inflammation, thromboembolism and progression to respiratory failure and disease severity (26–30), the role of sTREM-1 as a sepsis biomarker and mortality predictor (15, 16, 31, 32), or how upregulation of HGF, an anti-inflammatory cytokine, predicted ICU admission and death probability (17, 33, 34). It is worth mentioning that many of these investigations compared severe and non-severe, and hardly discriminated between mild and moderate COVID-19.

Importantly, the present study analyzes CRP, one of the first-identified and best-investigated biomarkers during the COVID-19 pandemics, together with the other above-mentioned biomarkers at hospital admission, and compares their efficiencies in predicting disease severity and mortality. Moreover, we also delignated the biomarkers’ profiles in two distinct groups of COVID-19 patients: Delta or predominantly-Omicron infections. First of all, CRP is normally lacking in viral infections; however, the macrophage activation syndrome which characterizes COVID-19 causes the pro-inflammatory hypercytokinemia profile which results in increasing the CRP synthesis by hepatocytes (11, 35). Elevated CRP levels lead to activation of endothelial cells and macrophages, stimulation of complement system *via* the classical route and inhibition of apoptosis of neutrophils, pathophysiological changes that exacerbate the vascular inflammation and thrombotic complication events which culminate with the cytokine storm (7, 11, 35). Indeed, recent studies have reported that elevated CRP together with higher D-Dimers and fibrinogen serum levels characterize the hyperinflammation and hypercoagulable states seen in severe and/or deceased COVID-19 patients (7, 36). Additional observational studies have reported higher CRP, NLR and LDH values in non-survivors, and their association with an increased risk of developing acute respiratory distress syndrome, cytokine release syndrome and sepsis (reviewed in (35). Importantly, several studies have found that high CRP levels (> 100 mg/L) were associated with higher odds for ventilator requirements (OR 2.5) and upgrade to ICU (OR 3.2), or even increased mortality rate (77.4% compared to 22.6% characterizing the group of cases with CRP < 100 mg/L) (8, 37). Interestingly, in a cohort study conducted on 2782 COVID-19 subjects, the serum CRP levels above 108 mg/L were associated with a 32.2% mortality rate (compared to only 17.8%, as seen for those with lower CRP values) and a 1.8-fold higher risk for disease severity (7). Importantly, high CRP levels associated with increased LDH and creatinine values as predictors for disease severity and death in other coronavirus infections, SARS in 2003 and MERS in 2012 (38–40). In influenza pneumonia (H1N1, H7N9), a high CRP value also correlated with disease severity, proving to be an independent predictor of mortality in the H7N9 infection, while leading to the cytokine storm characterized by elevated IL-6, MCP-1, and IP-10 levels (41, 42).

In our study we have shown that the mean serum CRP levels evolved from 12.59 mg/L in mild cases to 67.25 mg/L and 107.7 mg/L in moderate and severe patients, respectively. Among moderate cases, 25.4% had CRP values above 100 mg/L at hospital admission and survived, while the 2 deceased subjects had the CRP levels less than 100 mg/L. Among the severe cases, 39.4% of the COVID-19 subjects with high CRP (> 100 mg/L) died, while the mortality rate was only 14.3% among the cases with CRP less than 100 mg/L. In our study group (153 COVID-19 patients), the general mortality rate varied form 6.9% for CRP < 100 mg/L to 25.5% for cases with CRP > 100 mg/L. Importantly, the CRP values varied significantly between survivors (96.19 mg/L [95% CI 77.13-115.3]) and non-survivors (139.8 mg/L [95% CI 103.6-176], *p* = 0.0307), and a cut-off value of 79.12 mg/L yielded a sensitivity of 0.765 and specificity of 0.604 for mortality prediction. The CRP values further significantly correlated with immunological changes (marked increase of the pro-inflammatory biomarkers IL-1β, IL-6, MCP-1, with more pronounced changes in females than in male subjects) hematological abnormalities (increased WBC, neutrophilia, lymphopenia and eosinopenia with elevated NLR, PLR), hypercoagulation (increased D-Dimers, fibrinogen, and suPAR), and end-organ damage (high LDH, urea, AST).

Referring to hypercoagulation with venous thromboembolic events (VTE), recent studies have pointed out that the traditional markers such as D-Dimers and fibrinogen are less reliable than suPAR in predicting VTE in COVID-19 (30). The uPAR is a glycosylphosphatidylinositol-anchored membrane protein bound to immune cells (monocytes, macrophages and activated T cells), fibroblasts and endothelial cells. During inflammation, uPAR is cleaved and the soluble form, named suPAR, is released into circulation, and acting as a “damage-associated molecular pattern” (DAMP), it exerts local vascular inflammation or distal immune effects (26, 43). Elevated suPAR concentrations have been reported in other viral infections caused by hepatitis C and B viruses, HIV or Hantavirus, where it showed correlation with disease severity and mortality (44–47). SuPAR was also shown to be a discriminator of COVID-19 severity and a predictor of hospitalization time, findings which we have confirmed in our study (27–29). Additionally, we identified the mean serum values in mild, moderate and severe COVID-19 cases that followed a gradual increasing trend: 1.47 ng/mL, 3.68 ng/mL, and 5.06 ng/mL. Of interest, the suPAR levels showed the highest difference between Delta and Omicron cases: mean values of 5.21 ng/mL *vs* 2.09 ng/mL. Additionally, we have identified suPAR as a good independent discriminator between non-survivors and survivors, with a cut-off value of 5.2 ng/mL yielding a sensitivity of 0.765 and a specificity of 0.780. As the suPAR levels corroborate with cardiovascular diseases (CVD) (48), it is important to mention that no significant differences were notice in the CVD comorbidities among severe and non-severe COVID-19 subjects or survivors and non-survivors.

As an indirect readout of hyperinflammation, we further considered investigating the serum levels of anti-inflammatory molecules, such as sTREM-1 and HGF. Interestingly, the high CRP levels were also associated with the up-regulation of both these two biomarkers that play central roles in modulating inflammation. For instance, TREM-1 is a recently characterized pathogen recognition receptor (PRR) constitutively expressed on the surface of peripheral neutrophils and monocytes, but also by macrophages, epithelial and endothelial cells (31, 49, 50). Upon TLR signaling, its expression is up-regulated to further amplify the pro-inflammatory innate immune responses, by activating its downstream effectors such as PLCγ, MAPK and PI3K which promote the synthesis and release of inflammatory cytokines/chemokines (51, 52). During sepsis and hyperinflammation, TREM-1 activity is enhanced, leading to overactivation of macrophages that start to release metalloproteinases that are believed to proteolytically cleave the membrane TREM-1 and generate its soluble form, sTREM-1 (15, 53). During these pathological processes, sTREM-1 is believed to be additionally produced by macrophages and neutrophils as a splicing variant (54). Of interest, sTREM-1 act as a decoy for unknown TREM-1 ligands, thus counteracting the inflammatory cytokine release. Similarly, the synthesis of HGF, a pleiotropic cytokine, is up-regulated in macrophages and mesenchymal cells by pro-inflammatory cytokines such as IL-1β, TNF-α and IFN-γ (55–57). Importantly, HGF acts by promoting lung tissue repair through inhibiting the apoptosis of endothelial and epithelial cells, and plays a predominantly anti-inflammatory role by reducing IL-6 secretion at the expense of IL-10 production by macrophages (57–59). HGF has been previously shown to be also increased in severe cases of H1N1-induced pneumonia (60). Both sTREM-1 and HGF have been identified as predictors for disease severity and poor outcome in patients with COVID-19 (15–17, 31–34).

In our study we highlighted a gradual increase from mild to severe cases for both these two biomarkers, sTREM-1 and HGF. For instance, sTREM-1 mean values ranged from 147 pg/mL in mild cases to 289 pg/mL and 355 pg/mL in moderate and severe subjects, respectively. For HGF, the mean values from mild, to moderate, and severe were: 357 pg/mL, 819 pg/mL, and 1157 pg/mL. Importantly, sTREM-1 showed a very strong correlation with MCP-1 levels, while serum HGF strongly correlated with IL-1β in severe cases. They also showed significantly higher values in non-survivors compared to survivors (50% and 80% increase for sTREM-1 and HGF, respectively). While the sTREM-1 values were the same between the Delta and the predominantly-Omicron infected groups, the HGF values were significantly higher in the Delta group.

When comparing these inflammatory biomarkers for predicting the disease severity, the serum CRP levels at hospital admission best discriminated between severe and non-severe cases, followed by the LDH levels. For discriminating between Delta and Omicron infections, the suPAR levels showed the most prominent differences between these two variant infections with 64.3% of Delta cases with values over 4 ng/mL (the upper limit of the normal range) compared to only 5% of Omicron cases, observation which reassures the previous studies reporting lower rate of thromboembolic events in Omicron-infected individuals. Interestingly, when investigating the prediction capacity for poor outcome, LDH proved an excellent discrimination capacity, followed by HGF, sTREM-1 and suPAR in our study group.

As age might influence the serum levels of our investigated biomarkers (6), we further stratified the COVID-19 patients in two categories, younger or older than 60 years. Of all biomarkers, only sTREM-1 serum levels showed consistently higher values in the older groups of mild, moderate and severe subjects. We also noticed discrepancies between females and males: the females subjects showed more prominent significant changes with disease severity than male individuals. We could speculate that this is happening as males are known to exhibit higher basal inflammation and a weaker response against a stimulus, while displaying less efficient antioxidant mechanisms. These physiological differences might also contribute to the increased risk for males of developing severe forms of COVID-19 and a worse overall outcome, when compared with females. Nevertheless, the higher frequency of comorbidities observed in the severe group of subjects, including metabolic disorders and diseases of the circulatory system, might have contributed to the initial higher serum values of the here-in investigated inflammatory biomarkers.

Our study has also some limitations, including the sample size limited to 153 COVID-19 patients. However, the patient cohort was well-characterized due to the prospective design of the study, with proper review for all medical records and comorbidities, and assessment of clinical/paraclinical variables. Additionally, we should mention that not all the cases included in the Omicron group were confirmed by sequencing. Still, the predominantly-Omicron group comprised the cases admitted to the hospital in a time-frame when a general reduction in the Delta variant infections was nationally confirmed by the Romanian National Institute of Public Health (20, 21). Of note, larger cohort studies are needed to further confirm our described observations.

Overall, our data clearly state an important hyperinflammatory phenotype associated with disease severity characterized by enhanced serum levels of CRP, MCP-1, IL-1 and IL-6. When comparing this disease profile with the effect obtained upon vaccination, several differences were detected. For instance, in our previous study (22), while comparing the humoral responses following dual BNT162b2 vaccination in naïve or previously-infected SARS-CoV-2 individuals, a previous natural infectious status was associated with increased anti-SARS-CoV-2 antibody titers accompanied by increased IL-1 and IL-6, but lower levels of CRP and MCP-1. This serum profile reflects rather an effective polarization of the immune response towards Th2 with augmented specific-antibody generation, while minimizing the effects of the Th1 arm on cellular immunity, indirectly mirrored by reduced inflammation with lower CRP and MCP-1 levels following vaccination in primed subjects. Importantly, the severe cases of COVID-19 investigated in the present study associated a major increase in the CRP and MCP-1 serum levels assisted by enhanced suPAR, sTREM-1 and HGF values. The levels of these mentioned inflammation modulators were extremely higher in non-survivors and this molecular profile might also explain the coagulation abnormalities and the higher rate of hematological comorbidities (thrombocytopenia and anemia) characterizing these subjects at hospital admission. Starting from these observations, several questions emerge for potential studies to be tackled in future research. For instance, is it a stronger Th2 response, with humoral immunity assisted by a lower Th1-induced pro-inflammatory phenotype, beneficial for minimizing disease severity and mortality in SARS-CoV-2-infected patients? Preliminary reported data have indeed suggested that early treatment of COVID-19 with drugs targeting IL-1 (using an IL-1 receptor antagonist) or IL-6 (using humanized anti-IL-6 receptor monoclonal antibodies) proved to be beneficial, by reducing the mortality rate among severe cases of COVID-19 (11, 29, 30, 61). The retrospective analysis of our cohort of severe patients did not reveal significant improvement in the survival rate of those exposed to therapies targeting IL-6 (Tocilizumab) or IL-1 (Anakinra). However, the initial laboratory tests indicated a higher pro-inflammatory response in those patients compared to the subjects not exposed to such therapies, thus causing difficulties in achieving solid conclusions. It is most likely that the reported death rate among severe patients to be underestimated considering the expected beneficial effects of the applied anti-interleukin therapies. Importantly, no significant differences in the basal laboratory tests were observed among non-vaccinated or previously vaccinated subjects (which encountered only for 24%), in the context of a national vaccination rate of 40%.

To conclude, our data are of importance, as this is the first study to our knowledge that analyzes and compares CRP with the other 12 key predictors of disease severity and mortality, previously reported for COVID-19 (including suPAR, sTREM-1, HGF, MCP-1, LDH), while including mild, moderate and severe cases. Thus, we were able to identify that the CRP serum levels, among all investigated biomarkers, best discriminate between severe and non-severe subjects, while suPAR showed significant higher values in Delta-infections, and LDH proved to be an excellent predictor for mortality in COVID-19.

## Data availability statement

The original contributions presented in the study are included in the article/**Supplementary Material**. Further inquiries can be directed to the corresponding author.

## Ethics statement

The studies involving human participants were reviewed and approved by Institutional Ethics Committee - St Parascheva Clinical Hospital for Infectious Diseases, Iasi, Romania. The patients/participants provided their written informed consent to participate in this study.

## Author contributions

TP, MP-T and EGM conceptualized the study. TP, CEP, CGP, I-LM collected the samples and the relevant clinical information for each patient. MP-T and DC processed the samples. MP-T performed the initial statistical analysis with additional contribution from MM. TP, MP-T, PC and EGM analyzed the data. TP and MP-T prepared the initial draft which was reviewed and commented by all authors. All authors contributed to the article and approved the submitted version.
