# Supplementary material for: Comparison of C-reactive protein with distinct hyperinflammatory biomarkers in association with COVID-19 severity, mortality and SARS-CoV-2 variants
Source: Front Immunol. 2023 Jun 14;14:1213246. doi: 10.3389/fimmu.2023.1213246 (PMC10302717; doi:10.3389/fimmu.2023.1213246)
Supplement: Supplementary file 1 [file DataSheet_1.pdf]

Comparison of C-reactive protein with distinct hyperinflammatory biomarkers in association with COVID-19 severity, mortality and SARS-CoV-2 variants

Tudorita Gabriela Paranga, Mariana Pavel-Tanasa*, Daniela Constantinescu, Claudia Elena Plesca, Cristina Petrovici, Ionela-Larisa Miftode, Mihaela Moscalu, Petru Cianga, Egidia Miftode

*** Correspondence:**
Mariana Pavel-Tanasa, mariana.pavel-tanasa@umfiasi.ro

Supplementary materials contain:

Supplementary Figures 1-9
Supplementary Tables 1-12

Supplementary Figure 1

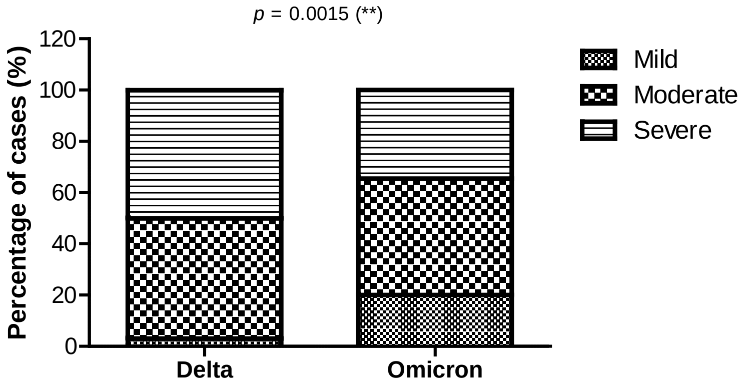

Supplementary Figure 1. Frequency of mild, moderate and severe cases in Delta and Omicron infections (**$p < 0.01$; chi-squared test).

Supplementary Figure 2

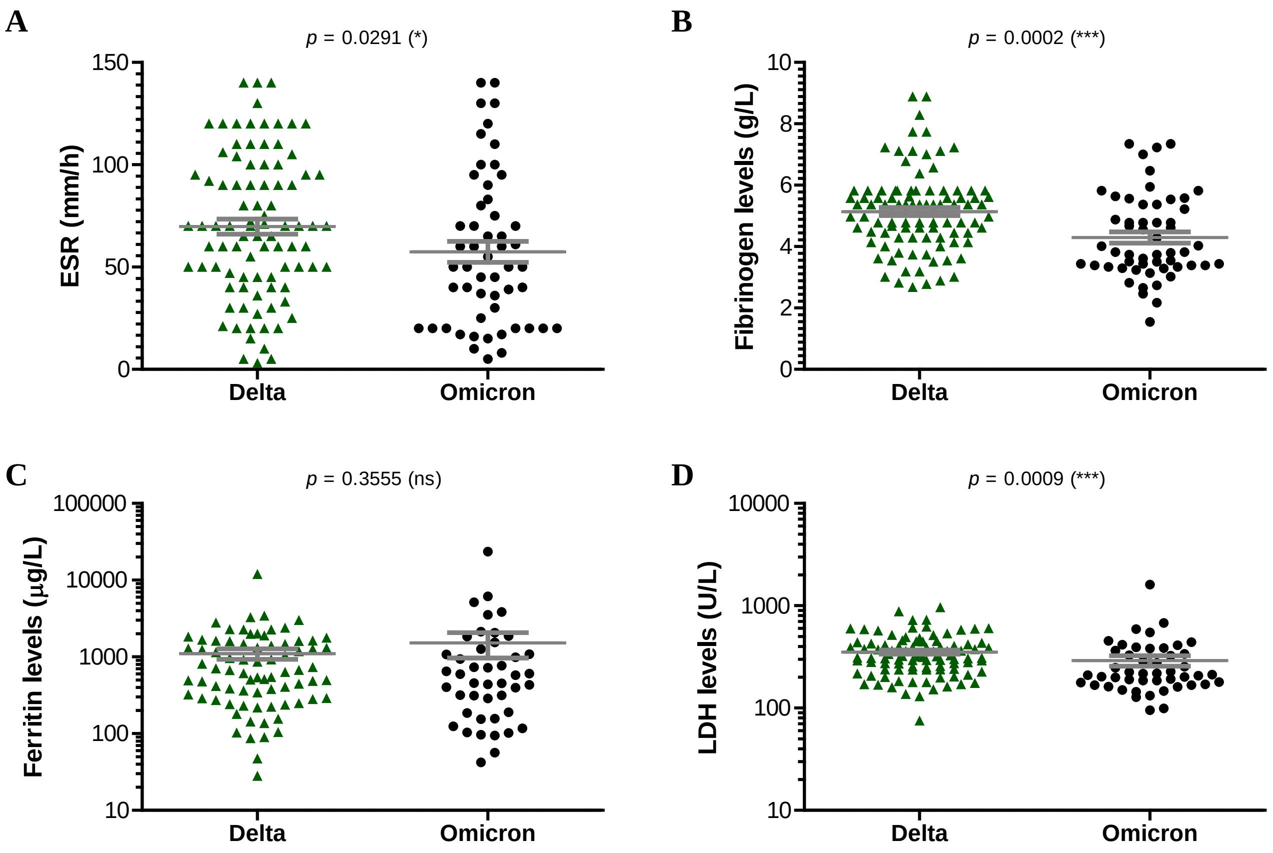

Supplementary Figure 2. Serum profile of common pro-inflammatory biomarkers in Delta and Omicron SARS-CoV-2 infections. (A) ESR, **(C)** fibrinogen, **(D)** ferritin, and **(E)** LDH levels for each category of SARS-CoV-2 infection: Delta or Omicron. The gray lines represent the mean ± SEM (***$p < 0.001$, *$p < 0.05$, ns – not significant; two-tailed Mann-Whitney test).

Supplementary Figure 3

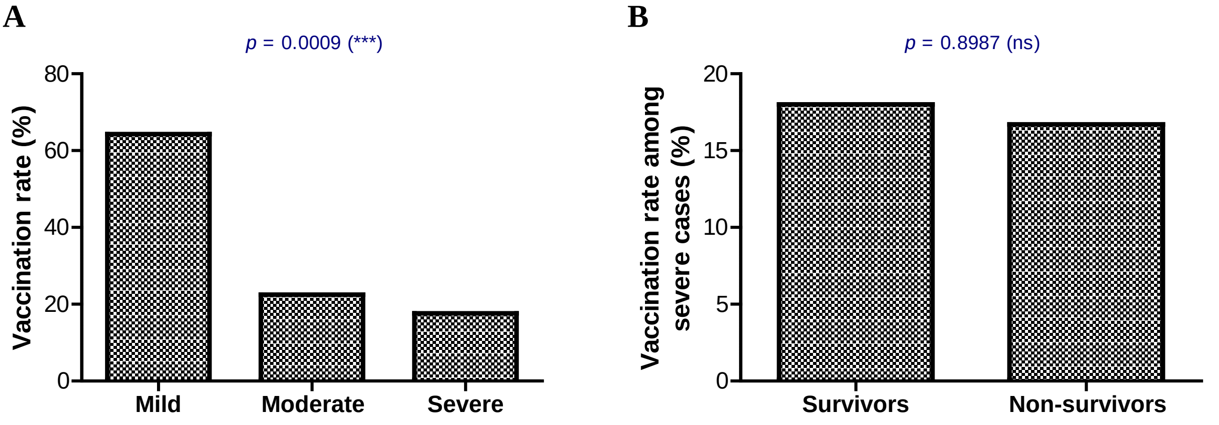

Supplementary Figure 3. Vaccination rate (A) for each category of COVID-19 severity (mild, moderate, severe), and **(B)** for survivors and non-survivors among severe cases (***$p < 0.001$, ns – not significant; chi-squared test).

Supplementary Figure 4

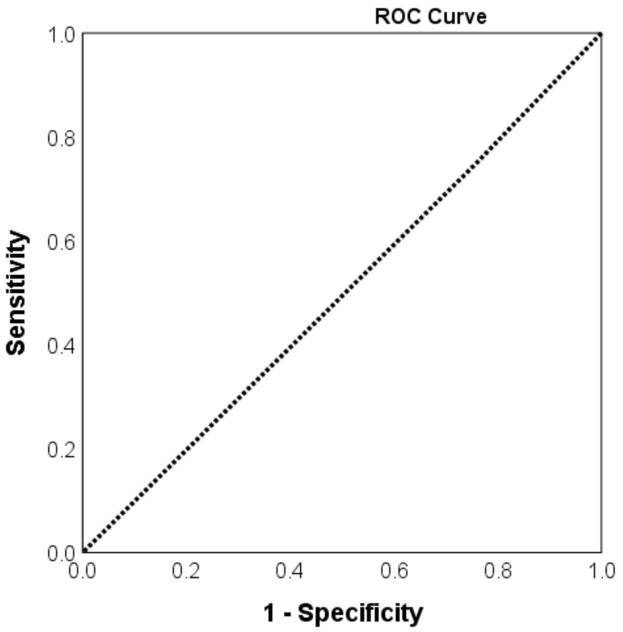

Variable	AUC	S.E.	*p*-value	95% CI
All cases	0.495	0.070	0.946	0.359-0.632
Severe cases	0.507	0.080	0.934	0.351-0.663

Supplementary Figure 4. ROC analysis generated for the association of vaccination status with mortality.

Supplementary Figure 5

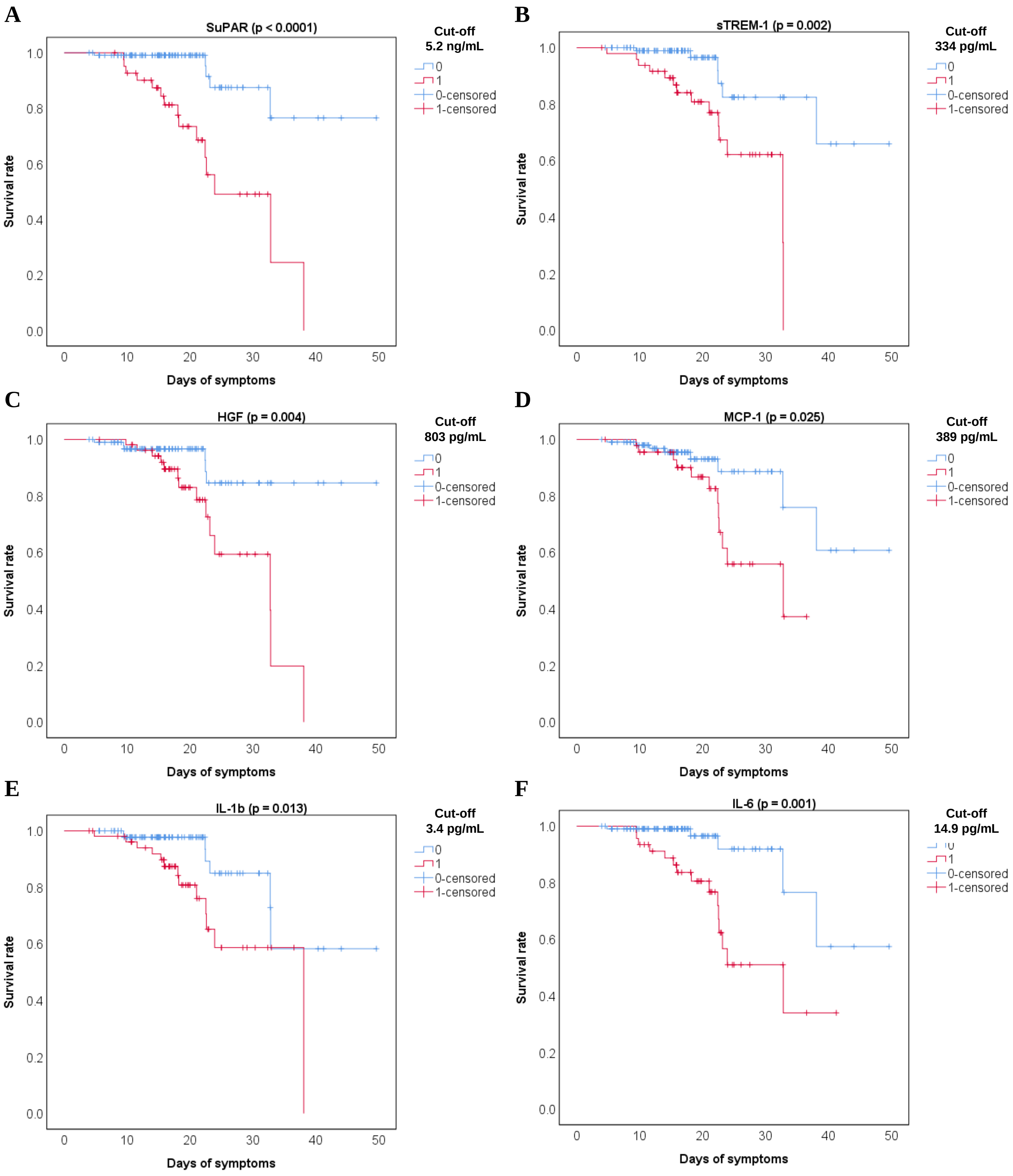

Supplementary Figure 5. Kaplan Meier survival curves for the indicated cut-off values of the following biomarkers: (A) suPAR, **(B)** sTREM-1, **(C)** HGF, **(D)** MCP-1, **(E)** IL-1β, and **(F)** IL-6. **0** = bellow cut-off value, **1** = above cut-off value (****$p < 0.0001$, **$p < 0.01$, *$p < 0.05$; Wald test).

Supplementary Figure 6

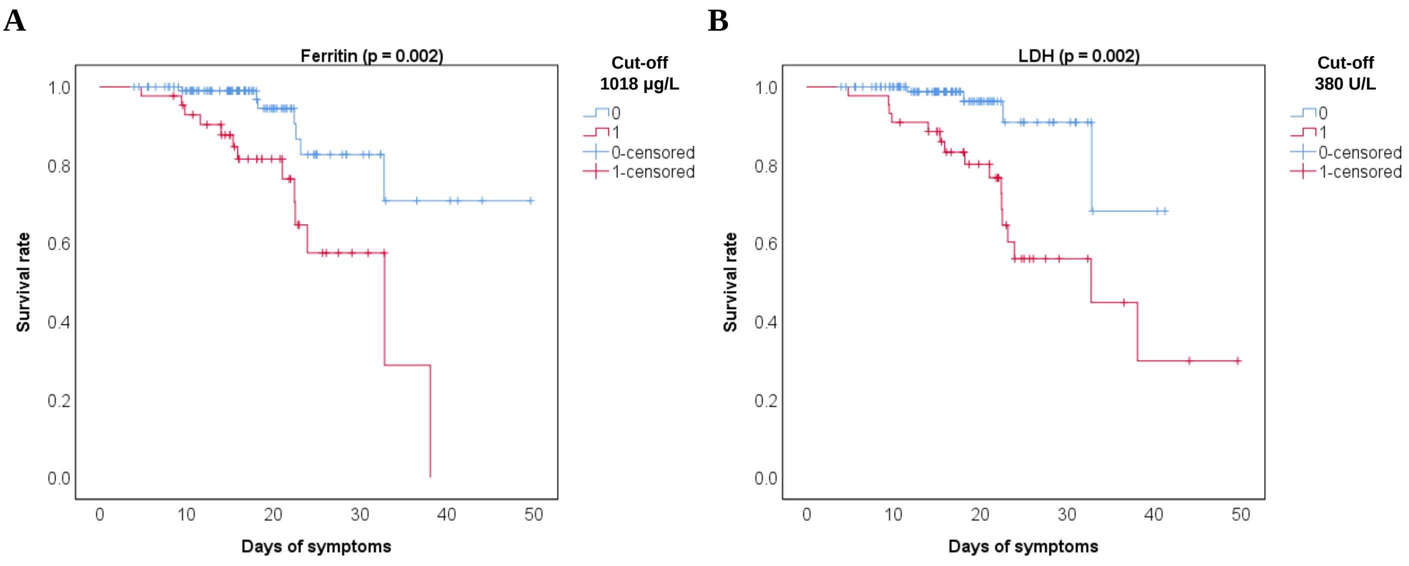

Supplementary Figure 6. Kaplan Meier survival curves for the indicated cut-off values of the following biomarkers: (A) ferritin, and **(B)** LDH. **0** = bellow cut-off value, **1** = above cut-off value (***p* < 0.01; Wald test).

Supplementary Figure 7

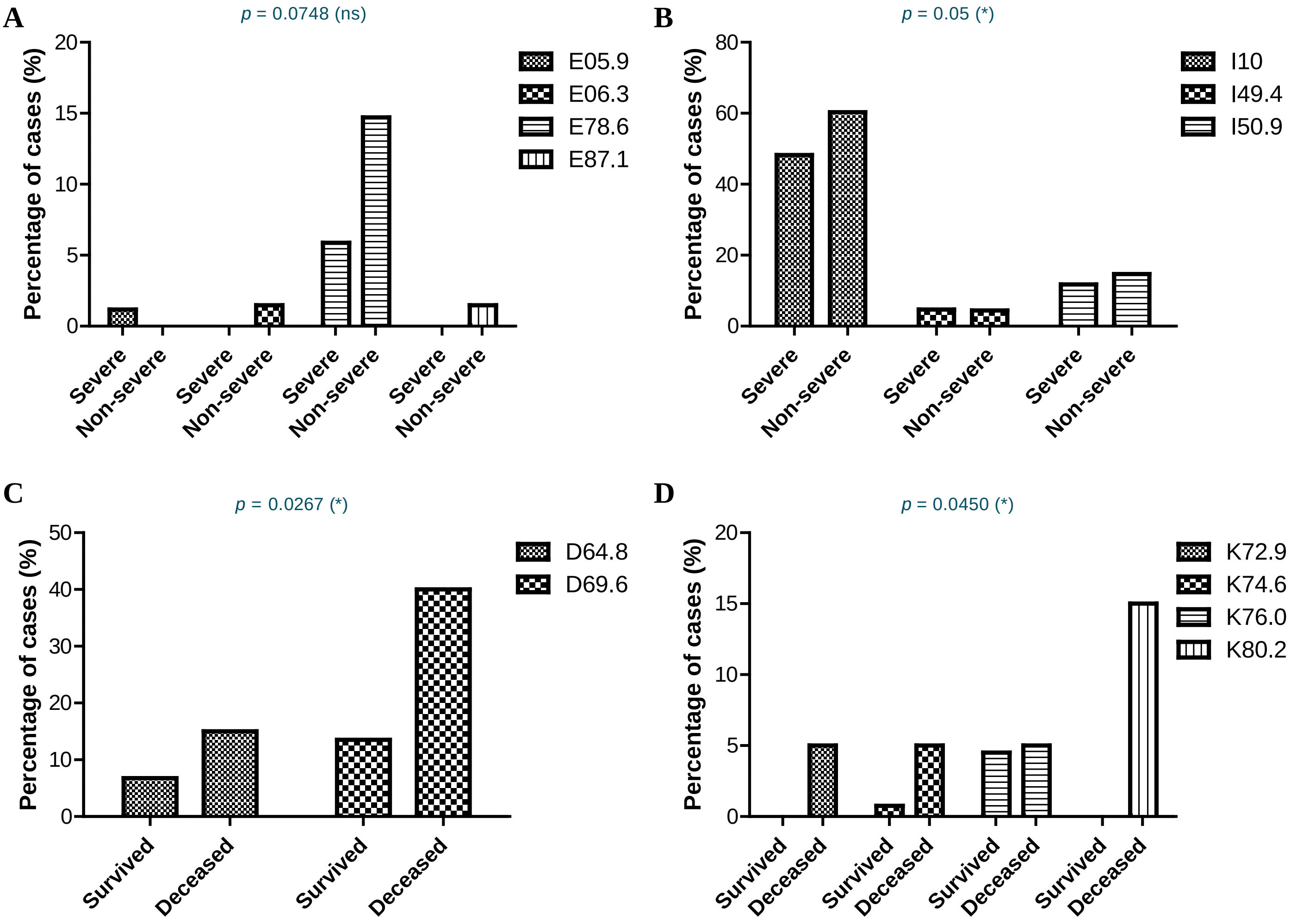

Supplementary Figure 7. Comorbidities associated with severe COVID-19 and mortality. Differences in the indicated comorbidity rates **(A-B)** between non-severe and severe COVID-19 patients, and **(C-D)** between survived and deceased COVID-19 patients (*$p < 0.05$, ns – not significant; chi-squared test).
E05.9 = Thyrotoxicosis, unspecified, E06.3 = Autoimmune thyroiditis, E78.6 = Lipoprotein deficiency, E87.1 = Hypo-osmolality and hyponatremia, I10 = Essential (primary) hypertension, I49.4 = Other and unspecified premature depolarization, I50.9 = Heart failure, unspecified, D64.8 = Other specified anemias, D69.6 = Thrombocytopenia, unspecified, K72.9 = Hepatic failure, unspecified, K74.6 = Other and unspecified cirrhosis of liver, K76.0 = Fatty (change of) liver, not elsewhere classified, K80.2 = Calculus of gallbladder without cholecystitis.

Supplementary Figure 8

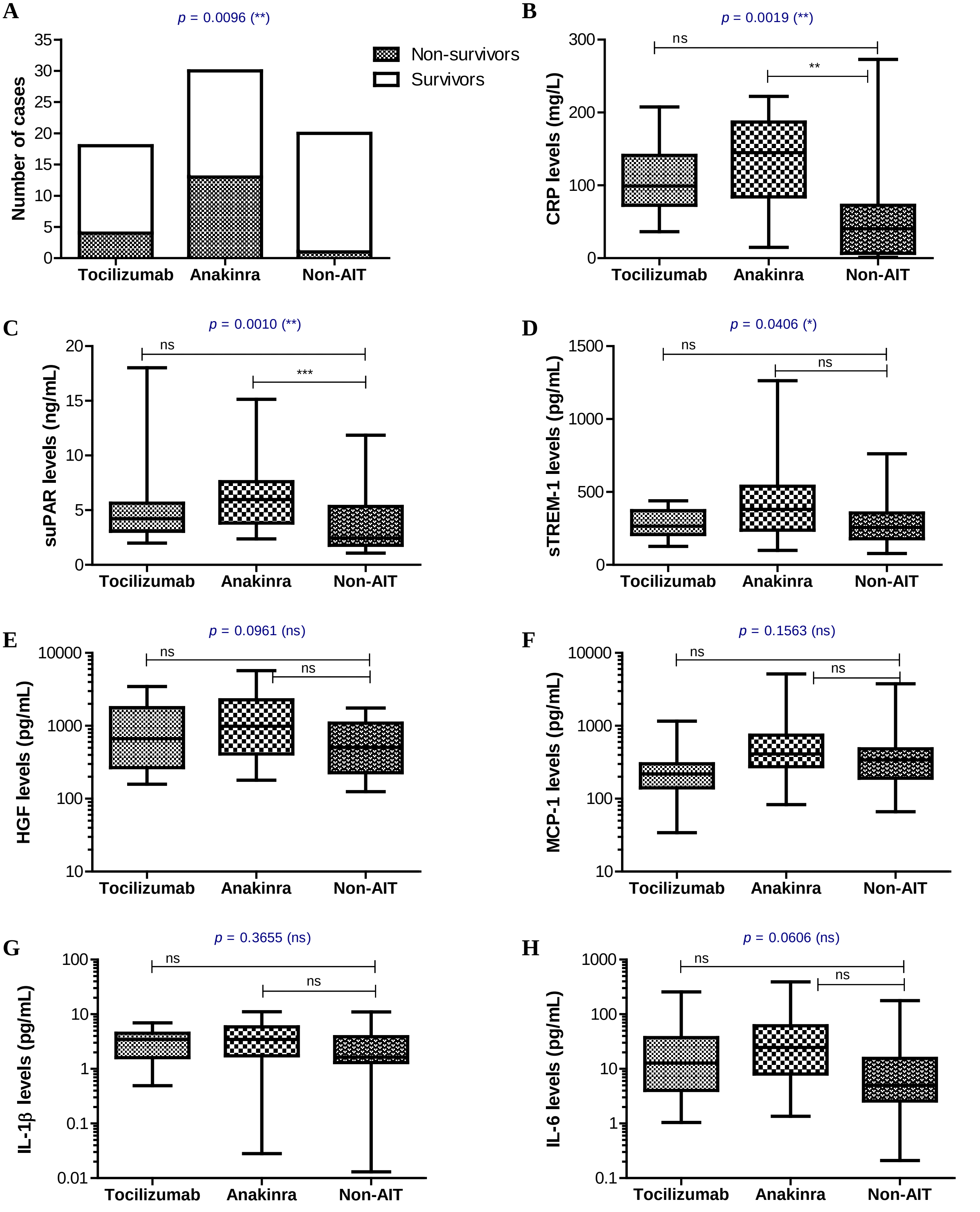

Supplementary Figure 8. Basal characteristics of severe cases receiving Tocilizumab, Anakinra or non-anti-interleukin medication. **(A)** Death rate for each treatment category (**$p < 0.01$; chi-squared test). Box and whiskers representation of **(B)** CRP, **(C)** suPAR, **(D)** sTREM-1, **(E)** HGF, **(F)** MCP-1, **(G)** IL-1β, **(H)** IL-6 serum levels for each treatment category (***$p < 0.001$, **$p < 0.01$, *$p < 0.05$, ns – not significant; Kruskal-Wallis statistics with Dunn's Multiple Comparison test).

Supplementary Figure 9

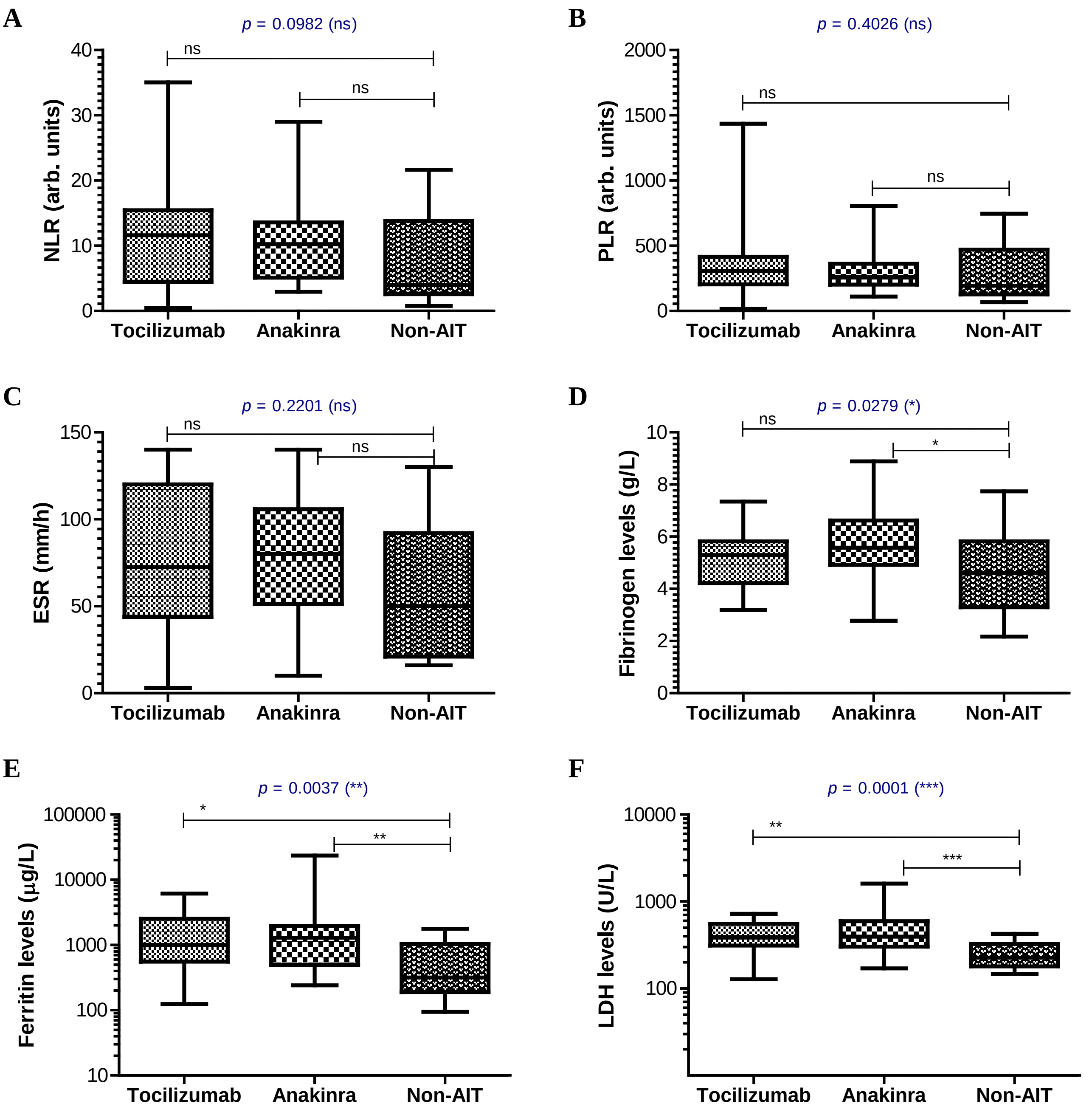

Supplementary Figure 9. Initial serum characteristics of severe cases receiving Tocilizumab, Anakinra or non-anti-interleukin medication. Box and whiskers representation of **(A)** NLR, **(B)** PLR, **(C)** ESR, **(D)** fibrinogen, **(E)** ferritin, and **(F)** LDH for each treatment category (***$p < 0.001$, **$p < 0.01$, *$p < 0.05$, ns – not significant; Kruskal-Wallis statistics with Dunn's Multiple Comparison test).

Supplementary Table 1. Patients' characteristics

	Mild	Moderate	Severe	*p*-value
**Cases (n, %)**<br>**F (n, %)**<br>**M (n, %)**	14 (9.2%)<br>9 (64.3%)<br>5 (35.7%)	71 (46.4%)<br>31 (43.7%)<br>40 (56.3%)	68 (44.4%)<br>25 (36.8%)<br>43 (63.2%)	0.1593
**Age (median, IQR)**	55 (45.5-66)	67 (57-73)	70 (57.5-79.5)	0.0385
**Mortality rate (n, %)**	0 (0%)	2 (2.8%)	18 (26.5%)	< 0.0001
**Hospitalisation days (median, IQR)**	6 (5-9.25)	10 (7-12)	13 (8-18)	< 0.0001
**Days of symptoms before hospitalisation (median, IQR)**	4.5 (2-6.5)	6.5 (4-10)	7 (4.5-9)	0.1712
**Vaccination (n, %)**	9 (64.3%)	16 (22.5%)	12 (17.6%)	0.0009

IQR, interquartile range; *p*, statistical significance coefficient.

Supplementary Table 2. Values of distinct biomarkers for each category of COVID-19 patients: mild, moderate and severe.

Analyte	Mild		Moderate		Severe		*p*-value	Normal range
	Median [IQR]	Mean [95% CI]	Median [IQR]	Mean [95% CI]	Median [IQR]	Mean [95% CI]		
CRP (mg/L)	7.01 [5.05-10.57]	12.59 [2.516-22.66]	54.14 [16.45-102.9]	67.25 [52.69-81.81]	99.18 [45.64-166]	107.7 [90.61-124.9]	**< 0.0001**	0-5
ESR (mm/h)	29.5 [16.5-53.75]	38.5 [20.6-56.4]	60.5 [40-80]	64.31 [55.97-72.66]	70 [42.5-102]	71.86 [62.53-81.2]	**0.0055**	0-20
Fibrinogen (g/L)	3.475 [3.215-4.08]	3.592 [3.248-3.936]	4.78 [3.74-5.37]	4.681 [4.404-4.958]	5.37 [4.29-5.82]	5.269 [4.907-5.63]	**< 0.0001**	2-4
Ferritin (µg/L)	213.6 [100.8-490.1]	408.4 [25.4-791.3]	498.2 [209.9-1261]	1034 [515.6-1553]	862.5 [353.3-1794]	1568 [779.1-2357]	**0.0029**	25-350
LDH (U/L)	168.5 [133.3-196.8]	166 [143.1-188.9]	280 [202.3-386]	294.3 [264.1-324.5]	338 [240-481]	396.4 [339.9-452.8]	**< 0.0001**	100-210
WBC (x 10^3/µl)	5.29 [4.80-6.21]	5.42 [4.57-6.27]	6.53 [4.79-7.8]	6.89 [6.16-7.63]	6.88 [4.97-11.33]	8.63 [7.45-9.8]	**0.0356**	6.0-13.5
Neutrophils (x 10^3/µl)	3.57 [2.69-4.35]	3.56 [2.68-4.43]	4.86 [3.4-5.94]	5.31 [4.67-5.95]	5.78 [3.53-9.57]	7.02 [5.9-8.14]	**0.0064**	1.5-8.0
Neutrophils (%)	62.5 [51.23-74.18]	63.81 [55.06-72.55]	77.3 [70.2-83.4]	75.65 [73.43-77.86]	84.9 [72.13-88.55]	79.39 [75.82-82.95]	**< 0.0001**	30.0-75.0
Eosinophils (x 10^3/µl)	0.015 [0-0.055]	0.049 [-0.006-0.103]	0 [0-0.02]	0.021 [0.012-0.03]	0 [0-0]	0.017 [0.005-0.029]	**0.0060**	0.0-0.5
Eosinophils (%)	0.30 [0-0.85]	1.01 [-0.25-2.27]	0 [0-0.3]	0.27 [0.15-0.39]	0 [0-0]	0.25 [0.07-0.43]	**0.0031**	0.0-5.0
Basophils (x 10^3/µl)	0.01 [0.01-0.02]	0.014 [0.011-0.016]	0.01 [0.01-0.02]	0.02 [0.015-0.021]	0.01 [0.01-0.02]	0.01 [0.012-0.018]	0.3320	0.0-0.2
Basophils (%)	0.25 [0.2-0.33]	0.26 [0.2-0.32]	0.2 [0.1-0.3]	0.27 [0.22-0.33]	0.2 [0.1-0.3]	0.21 [0.17-0.26]	0.1361	0.0-2.0
Lymphocytes (x 10^3/µl)	1.23 [0.83-1.66]	1.29 [0.94-1.64]	1.02 [0.66-1.32]	1.06 [0.94-1.18]	0.79 [0.58-0.96]	1.01 [0.63-1.4]	**0.0018**	3.0-8.0
Lymphocytes (%)	24.8 [15.38-38.48]	25.36 [18.43-32.29]	15.4 [11.3-21.3]	16.61 [14.77-18.45]	8.75 [6.6-19.73]	13.31 [10.64-15.97]	**< 0.0001**	20.0-45.0
Monocytes (x 10^3/µl)	0.5 [0.28-0.6]	0.5 [0.34-0.66]	0.42 [0.27-0.6]	0.44 [0.41-0.55]	0.34 [0.23-0.63]	0.54 [0.34-0.75]	0.4624	0.0-1.0
Monocytes (%)	9.5 [7.33-11.38]	9.57 [6.86-12.28]	6.4 [4.8-8.7]	7.09 [6.37-7.82]	4.8 [3.28-7.4]	6.85 [4.73-8.97]	**0.0014**	0.0-12.0
Hemoglobin (g/dL)	13.45 [10.88-15.53]	12.95 [11.51-14.39]	13.3 [12-14.1]	12.86 [12.41-13.32]	13.65 [12.33-14.68]	13.6 [13.17-14.03]	0.1605	12.0-15.0
Hematocrit (%)	39.05 [32.15-44.08]	37.43 [33.37-41.49]	38.3 [34.8-40.5]	37.41 [36.14-38.67]	38.65 [36.28-43.25]	39.26 [38.08-40.45]	0.2735	32.0-42.0
Platelets (x 10^3/µl)	193 [139.8-246]	212.4 [157-267.9]	206 [152-280]	229.9 [204.5-255.4]	190.5 [143.8-293.8]	227.4 [200.2-254.7]	0.8235	150-380
Total serum protein (g/L)	76.2 [67.31-77.53]	73.16 [68.55-77.77]	70.98 [67.22-76.17]	70.39 [68.25-72.53]	70.46 [63.93-74.46]	69.53 [67.48-71.57]	0.272	67-87
AST (U/L)	27 [21-37.3]	39.7 [17.9-61.5]	40 [25-55]	46.8 [39.3-54.3]	44 [29-75]	74.1 [36.4-111.7]	**0.0167**	5-31
ALT (U/L)	27.5 [20.5-48.8]	43.6 [22-65.3]	32 [18-49]	40.1 [32.6-47.5]	37 [23.3-64]	64.6 [36-93.2]	0.264	5-31
Total bilirubin (mg/dL)	0.44 [0.265-0.765]	0.56 [0.298-0.817]	0.45 [0.33-0.63]	0.49 [0.434-0.543]	0.48 [0.36-0.63]	0.54 [0.459-0.617]	0.7032	0.2-1
Prothrombin index	88.2 [74.5-99.5]	85 [70.1-100]	81.7 [74.6-93.5]	82 [76.3-87.7]	81.1 [70.9-91.8]	79 [73.9-84.2]	0.4746	78-126
D-dimer (µg/mL)	0.39 [0.33-0.55]	0.5 [0.27-0.73]	0.51 [0.40-0.67]	0.87 [0.51-1.23]	0.68 [0.50-1.04]	1.24 [0.79-1.7]	**0.0011**	0-0.5
Blood glucose (mg/dL)	131 [95.75-265.3]	173.4 [111.7-235.1]	128 [104-171]	146.3 [131.7-161]	137.5 [114.8-181.5]	161.1 [143.8-178.3]	0.3606	70-115
Urea (mg/dL)	31 [25.5-51.25]	37.21 [26.45-47.98]	41 [29-58]	48.72 [41.37-56.06]	49.5 [35.5-69.75]	58.29 [48.79-67.8]	**0.0156**	15-50
Creatinine (mg/dL)	0.91 [0.84-1.14]	0.97 [0.87-1.07]	0.96 [0.85-1.14]	1.12 [0.97-1.27]	1.04 [0.83-1.29]	1.17 [1.03-1.3]	0.3804	0.5-0.9
Potassium (mmol/L)	3.87 [3.73-4.33]	3.96 [3.66-4.25]	4.14 [3.84-4.43]	4.13 [4.02-4.25]	4.26 [3.9-4.7]	4.23 [4.09-4.37]	0.095	3.7-5.3
Sodium (mmol/L)	142.7 [140.2-143.5]	141.3 [138.6-144.0]	141.2 [139.8-142.9]	141.1 [140.5-141.8]	140 [137.9-141.9]	139.9 [139.3-140.6]	**0.0047**	135-148
Ionized calcium (mg/dL)	4.76 [4.68-4.88]	4.64 [4.24-5.04]	4.6 [4.36-4.92]	4.65 [4.55-4.75]	4.66 [4.4-4.91]	4.62 [4.52-4.72]	0.4484	4-5.6
Chloride (mmol/L)	101 [98.8-103.1]	100.2 [97.8-102.6]	100.7 [99.1-102.4]	100.5 [99.8-101.1]	99.7 [98.1-101.5]	99.6 [99-100.3]	0.0917	98-109

CRP, C-reactive protein; ESR, erythrocyte sedimentation rate; LDH, lactate dehydrogenase; WBC, white blood cells; AST, aspartate transaminase; ALT, alanine transaminase; IQR, interquartile range; CI, confidence interval; *p*, statistical significance coefficient.

Supplementary Table 3. The initial values of inflammatory biomarkers for each category of severe COVID-19 patients: survivors or non-survivors.

Analyte	Survivors		Non-survivors		*p*-value
	Median [IQR]	Mean [95% CI]	Median [IQR]	Mean [95% CI]	
**CRP (mg/L)**	88.4 [40.66-154.7]	96.19 [77.13-115.3]	143.8 [80.87-208.1]	139.8 [103.6-176]	**0.0307**
**suPAR (ng/mL)**	3.83 [2.46-5.60]	4.48 [3.62-5.35]	6.47 [5.42-8.30]	6.66 [5.17-8.15]	**0.0018**
**sTREM-1 (pg/mL)**	279.3 [207.2-420]	312.9 [267.5-358.4]	405.8 [304.1-645.2]	470.5 [331.9-609.1]	**0.0189**
**HGF (pg/mL)**	508.2 [257.6-1423]	950.6 [670-1231]	1248 [692.3-2407]	1727 [997.2-2457]	**0.0078**
**MCP-1 (pg/mL)**	301.4 [187-478.5]	519.6 [301.7-737.5]	445.1 [299.3-837.9]	952.5 [297.7-1607]	**0.0121**
**IL-1β (pg/mL)**	2.49 [1.30-4.63]	3.11 [2.39-3.83]	3.64 [2.49-5.85]	4.70 [3.38-6.02]	**0.0154**
**IL-6 (pg/mL)**	9.02 [2.59-25.36]	38.53 [14.56-62.49]	40.95 [24.28-94.58]	67.63 [32.15-103.1]	**0.0014**
**NLR**	8.61 [3.35-12.85]	9.38 [7.44-11.33]	10.89 [4.93-20.39]	12.87 [8.17-17.57]	0.1002
**PLR**	289 [180-421]	313.1 [262.3-363.8]	227.2 [176.3-356.3]	368.3 [191.1-545.6]	0.4005
**ESR (mm/h)**	65 [36-100]	68.87 [57.14-80.6]	80 [50-105.3]	79.67 [64.47-94.86]	0.3049
**Fibrinogen (g/L)**	5.37 [3.92-6.17]	5.21 [4.75-5.66]	5.48 [4.71-5.69]	5.44 [4.86-6.01]	0.5770
**Ferritin (μg/L)**	703 [304.3-1623]	1580 [512-2648]	1429 [746.6-1966]	1534 [1033-2035]	**0.0324**
**LDH (U/L)**	310 [210.5-417.5]	367.1 [295.5-438.7]	422.5 [389.5-594.5]	475.9 [400.6-551.3]	**0.0012**

CRP, C-reactive protein; suPAR, soluble urokinase plasminogen activator receptor; sTREM-1, soluble triggering receptor expressed on myeloid cells-1; HGF, hepatocyte growth factor; MCP-1, monocyte chemoattractant protein-1; IL-1β, interleukin-1 beta; IL-6, interleukin-6; NLR, neutrophil-lymphocyte ratio; PLR, platelet-lymphocyte ratio; ESR, erythrocyte sedimentation rate; LDH, lactate dehydrogenase; *p*, statistical significance coefficient.

Supplementary Table 4. Basal laboratory characteristics of vaccinated and non-vaccinated COVID-19 patients (mild, moderate, and severe cases).

Vaccination	Mild			Moderate			Severe		
	Yes	No	*p-value*	Yes	No	*p-value*	Yes	No	*p-value*
**Variable, median [IQR]**									
CRP (mg/L)	6.81 [3.72-17.69]	7.2 [5.81-10.86]	1.0000	33.87 [17.71-83.92]	54.35 [14.38-121.50]	0.7958	148.1 [68.98-177.50]	95.44 [40.26-164.40]	0.2726
suPAR (mg/mL)	1.12 [0.95-1.79]	1.72 [1.33-1.86]	0.2977	2.82 [1.42-4.55]	3.35 [2.21-4.99]	0.2081	4.21 [2.73-6.24]	4.31 [2.47-6.23]	0.8539
sTREM-1 (pg/mL)	144.5 [118.6-184.9]	146.0 [86.8-199.5]	1.0000	247.1 [217.0-513.1]	252.8 [193.7-334.1]	0.2204	345.7 [182.5-505.4]	307.2 [213.7-457.6]	0.9705
HGF (pg/mL)	318.7 [194.3-714.6]	248.0 [55.5-541.8]	0.4762	598.9 [275.5-1863.0]	470.2 [247.3-991.9]	0.4681	532.9 [280.6-1734.0]	859.3 [313.1-1738.0]	0.6774
MCP-1 (pg/mL)	214.4 [106.4-409.6]	268.2 [219.0-315.0]	0.7619	195.6 [105.6-326.6]	285.1 [208.3-528.9]	**0.0240**	479.4 [304.8-1663.0]	308.9 [200.5-518.6]	0.0744
IL-1 (pg/mL)	1.59 [1.21-2.36]	0.54 [0.03-1.30]	0.0691	2.79 [1.13-5.54]	2.49 [1.30-3.64]	0.4705	3.07 [0.95-3.92]	3.25 [1.30-5.44]	0.3671
IL-6 (pg/mL)	1.75 [0.4-5.02]	1.83 [0.13-6.67]	1.0000	5.65 [2.59-10.13]	5.65 [2.59-18.69]	0.7901	44.78 [14.31-138.60]	9.75 [2.99-37.08]	**0.0218**
NLR	2.68 [2.24-3.77]	1.35 [0.95-8.76]	0.5185	5.15 [2.85-10.37]	5.10 [3.37-7.20]	0.9276	11.29 [3.12-17.22]	8.39 [3.42-12.93]	0.5965
PLR	157.6 [124.9-195.7]	135.0 [110.2-451.4]	1.0000	305.2 [131.2-547.0]	211.0 [146.4-304.8]	0.2158	331.2 [157.5-528.4]	256.9 [178.8-398.1]	0.5904
ESR (mm/h)	20 [19-45]	39 [12-100]	0.7886	60 [39-78]	65 [40-80]	0.8555	100 [38-120]	70 [45-98]	0.3131
Fibrinogen (g/L)	3.29 [2.98-4.28]	3.51 [3.42-3.95]	0.6064	4.30 [3.47-5.37]	4.78 [3.96-5.42]	0.1996	5.54 [4.405-7.23]	5.37 [4.29-5.82]	0.2476
Ferritin (µg/L)	190.5 [96.4-403.7]	596.7 [102.2-1843.0]	0.2667	312.5 [116.9-2065.0]	539.3 [280.7-1259.0]	0.4259	1455.0 [685.7-4176.0]	811.4 [321.5-1621.0]	0.0958
LDH (U/L)	149.5 [130.5-186.8]	184.5 [167.8-208.0]	0.2141	236.0 [186-399.8]	292.0 [206.0-386.0]	0.4251	412.0 [237.5-531.5]	327.0 [240.0-446.0]	0.5534
Age (years)	59 [46-69]	54 [40-64]	0.5045	70 [66-78]	65 [55-73]	**0.0280**	74 [61-82]	70 [57-78]	0.4291
Onset of symptoms before hospital admission (days)	4.0 [1.5-5.5]	6.0 [2.5-12]	0.6993	6.0 [3.3-7.0]	7.0 [4.0-10.0]	0.1248	6.0 [3.5-9.0]	7.0 [5.0-9.75]	0.5719

CRP, C-reactive protein; suPAR, soluble urokinase plasminogen activator receptor; sTREM-1, soluble triggering receptor expressed on myeloid cells-1; HGF, hepatocyte growth factor; MCP-1, monocyte chemoattractant protein-1; IL-1β, interleukin-1 beta; IL-6, interleukin-6; NLR, neutrophil-lymphocyte ratio; PLR, platelet-lymphocyte ratio; ESR, erythrocyte sedimentation rate; LDH, lactate dehydrogenase; IQR, interquartile range; *p*, statistical significance coefficient.

Supplementary Table 5. Statistical evaluation for the indicated prediction models related to Figure 8B.

Mathematical model	AUC	S.E.	*p*-value	95% Confidence Interval
**Model 1_1 (CRP_suPAR_sTREM-1_HGF_IL-6_NLR_PLR_ESR_Fibrinogen_Ferritin_LDH)**	0.799	0.042	< 0.0001	0.717-0.881
**Model 1_2 (CRP_suPAR_sTREM-1_HGF_IL-6)**	0.763	0.045	< 0.0001	0.674-0.852
**Model 1_3 (CRP_NLR_PLR_ESR_Fibrinogen_Ferritin_LDH)**	0.769	0.045	< 0.0001	0.680-0.857
**Model 1_4 (CRP_suPAR_Fibrinogen_LDH)**	0.766	0.045	< 0.0001	0.678-0.855
**Model 1_5 (CRP_suPAR)**	0.744	0.047	< 0.0001	0.652-0.836
**Model 1_6 (CRP_sTREM-1)**	0.738	0.048	< 0.0001	0.644-0.831
**Model 1_7 (CRP_HGF)**	0.731	0.048	< 0.0001	0.638-0.825
**Model 1_8 (CRP_IL-6)**	0.743	0.047	< 0.0001	0.650-0.835
**Model 1_9 (CRP_Fibrinogen)**	0.723	0.049	< 0.0001	0.628-0.818
**Model 1_10 (CRP_LDH)**	0.744	0.047	< 0.0001	0.652-0.837

CRP, C-reactive protein; suPAR, soluble urokinase plasminogen activator receptor; sTREM-1, soluble triggering receptor expressed on myeloid cells-1; HGF, hepatocyte growth; IL-6, interleukin-6; NLR, neutrophil-lymphocyte ratio; PLR, platelet-lymphocyte ratio; ESR, erythrocyte sedimentation rate; LDH, lactate dehydrogenase; AUC, area under curve; *p*, statistical significance coefficient.

Supplementary Table 6. Univariate and multivariate regression analysis of paraclinical cut-off values and clinical variables in relation to disease severity.

**Variable (Severe disease)**	**Univariate analysis**			**Multivariate analysis**		
	**HR**	**95% CI**	***p*-value**	**HR**	**95% CI**	***p*-value**
CRP (mg/L)	4.37	2.21-8.65	**< 0.0001**	2.96	1.27-6.88	**0.012**
suPAR (mg/mL)	3.09	1.59-6.00	**0.001**	1.42	0.62-3.24	0.409
sTREM-1 (pg/mL)	2.71	1.40-5.23	**0.003**	0.82	0.34-1.95	0.649
HGF (pg/mL)	2.40	1.22-4.71	**0.011**	1.39	0.60-3.19	0.443
IL-6 (pg/mL)	2.91	1.50-5.64	**0.002**	1.75	0.78-3.92	0.173
NLR	5.91	2.84-12.33	**< 0.0001**	7.85	2.67-23.09	**< 0.0001**
PLR	2.37	1.23-4.54	**0.010**	1.00	0.99-1.00	0.236
Age (> 60 years)	1.53	0.80-2.93	0.202	0.96	0.41-2.25	0.926
Gender (M)	1.45	0.73-2.87	0.286	1.01	0.46-2.23	0.973
Vaccination (no)	0.51	0.24-1.12	0.094	0.42	0.15-1.12	0.083

CRP, C-reactive protein; suPAR, soluble urokinase plasminogen activator receptor; sTREM-1, soluble triggering receptor expressed on myeloid cells-1; HGF, hepatocyte growth factor; IL-6, interleukin-6; NLR, neutrophil-lymphocyte ratio; PLR, platelet-lymphocyte ratio; HR, hazard ratio; CI, confidence interval; *p*, statistical significance coefficient.

Supplementary Table 7. Statistical evaluation for the indicated prediction models related to Figure 8C.

Mathematical model	AUC	S.E.	*p*-value	95% Confidence Interval
**Model 2_1 (suPAR_HGF_IL-6_ESR_Fibrinogen_LDH)**	0.932	0.023	< 0.0001	0.888-0.977
**Model 2_1 (suPAR_HGF_IL-6)**	0.918	0.025	< 0.0001	0.869-0.967
**Model 2_1 (suPAR_ESR_Fibrinogen_LDH)**	0.925	0.024	< 0.0001	0.878-0.972
**Model 2_1 (suPAR_HGF)**	0.914	0.026	< 0.0001	0.863-0.964
**Model 2_1 (suPAR_IL-6)**	0.917	0.025	< 0.0001	0.868-0.967
**Model 2_1 (suPAR_ESR)**	0.913	0.026	< 0.0001	0.862-0.964
**Model 2_1 (suPAR_Fibrinogen)**	0.911	0.026	< 0.0001	0.86-0.962
**Model 2_1 (suPAR_LDH)**	0.926	0.024	< 0.0001	0.879-0.972

Abbreviations: suPAR = soluble urokinase plasminogen activator receptor; HGF = hepatocyte growth; IL-6 = interleukin-6; ESR = erythrocyte sedimentation rate; LDH = lactate dehydrogenase; AUC = area under curve; *p* = statistical significance coefficient.

Supplementary Table 8. Univariate and multivariate regression analysis of paraclinical cut-off values and clinical variables in relation to Delta variant infections.

Variable (Delta variant)	Univariate analysis			Multivariate analysis		
	**HR**	**95% CI**	***p*-value**	**HR**	**95% CI**	***p*-value**
suPAR (mg/mL)	30.83	11.03-86.21	**< 0.0001**	41.59	10.19-169.80	**0.000**
HGF (pg/mL)	2.76	1.40-5.47	**0.004**	1.57	0.53-4.61	0.412
IL-6 (pg/mL)	5.86	2.52-13.72	**< 0.0001**	4.45	1.27-15.64	**0.020**
ESR (mm/h)	1.89	0.95-3.77	0.069	0.85	0.29-2.51	0.773
Fibrinogen (g/L)	5.72	2.69-12.14	**< 0.0001**	2.81	0.82-9.68	0.101
LDH (U/L)	4.83	2.37-9.86	**< 0.0001**	0.64	0.19-2.11	0.461
Severe (yes)	1.90	0.96-3.75	0.066	0.55	0.19-1.64	0.283
Age (> 60 years)	1.04	0.52-2.09	0.913	0.63	0.21-1.88	0.406
Gender (M)	0.85	0.44-1.67	0.642	0.29	0.09-0.86	**0.025**
Vaccination (no)	0.27	0.13-0.59	**0.001**	0.20	0.06-0.64	**0.006**

suPAR, soluble urokinase plasminogen activator receptor; HGF, hepatocyte growth factor; IL-6, interleukin-6; ESR, erythrocyte sedimentation rate; LDH, lactate dehydrogenase; HR, hazard ratio; CI, confidence interval; *p*, statistical significance coefficient.

Supplementary Table 9. Statistical evaluation for the indicated prediction models related to Figure 8D.

Mathematical model	AUC	S.E.	*p*-value	95% Confidence Interval
**Model 3_1 (LDH_CRP_suPAR_sTREM-1_HGF_MCP-1_IL-1β_IL-6_NLR_Ferritin)**	0.869	0.048	< 0.0001	0.775-0.963
**Model 3_2 (LDH_CRP_suPAR_sTREM-1_HGF_MCP-1_IL-1β_IL-6)**	0.808	0.057	< 0.0001	0.696-0.92
**Model 3_3 (LDH_NLR_Ferritin)**	0.846	0.046	< 0.0001	0.755-0.936
**Model 3_4 (LDH_suPAR_sTREM-1_HGF_IL-1β)**	0.851	0.042	< 0.0001	0.769-0.933
**Model 3_5 (LDH_CRP)**	0.742	0.062	0.002	0.620-0.864
**Model 3_6 (LDH_suPAR)**	0.800	0.050	< 0.0001	0.702-0.897
**Model 3_7 (LDH_HGF)**	0.836	0.044	< 0.0001	0.750-0.922
**Model 3_8 (LDH_IL-1β)**	0.830	0.044	< 0.0001	0.743-0.917

LDH, lactate dehydrogenase; CRP, C-reactive protein; suPAR, soluble urokinase plasminogen activator receptor; sTREM-1, soluble triggering receptor expressed on myeloid cells-1; HGF, hepatocyte growth; MCP-1, monocyte chemoattractant protein-1; IL-1β, interleukin-1 beta; IL-6, interleukin-6; NLR, neutrophil-lymphocyte ratio; AUC, area under curve; *p*, statistical significance coefficient.

Supplementary Table 10. Statistical evaluation for the indicated prediction models for mortality in severe COVID-19 subjects.

Mathematical model	AUC	S.E.	*p*-value	95% Confidence Interval
**CRP (mg/L)**	0.656	0.081	0.058	0.497-0.814
**SuPAR (ng/mL)**	0.748	0.073	0.003	0.605-0.892
**sTREM-1 (pg/mL)**	0.694	0.078	0.018	0.542-0.846
**HGF (pg/mL)**	0.720	0.067	0.008	0.589-0.851
**MCP-1 (pg/mL)**	0.707	0.070	0.012	0.569-0.845
**IL-1β (pg/mL)**	0.699	0.066	0.016	0.570-0.828
**IL-6 (pg/mL)**	0.763	0.068	0.001	0.630-0.896
**Model 4_1 (CRP_suPAR_sTREM-1_HGF_MCP-1_IL-1β_IL-6)**	0.757	0.070	0.002	0.621-0.894
**Model 4_2 (Model 4_1 and comorbidities)**	0.824	0.054	< 0.0001	0.717-0.930

CRP, C-reactive protein; suPAR, soluble urokinase plasminogen activator receptor; sTREM-1, soluble triggering receptor expressed on myeloid cells-1; HGF, hepatocyte growth; MCP-1, monocyte chemoattractant protein-1; IL-1β, interleukin-1 beta; IL-6, interleukin-6; AUC, area under curve; *p*, statistical significance coefficient.

Supplementary Table 11. Multivariate regression analysis of survival rate for the indicated applied therapies.

Variable	Multivariate analysis		
	OR	95% CI	*p*-value
Tocilizumab vs. non-AIT	0.163	0.012-2.149	0.168
Anakinra vs. non-AIT	0.079	0.007-0.851	**0.036**
Remdesivir (Yes/ No)	0.317	0.072-1.393	0.128
Aspirin (Yes/ No)	0.538	0.044-6.615	0.628
Diclofenac (Yes/ No)	0.701	0.106-4.659	0.713
Metamizole (Yes/ No)	5E7	-	0.995
Acetaminophen (Yes/ No)	0.568	0.107-3.023	0.508

OR, odds ratio; CI, confidence interval; *p*, statistical significance coefficient.

Supplementary Table 12. Statistical evaluation of baseline characteristics of severe COVID-19 patients receiving anti-inflammatory therapy.

**Baseline characteristics of severe patients**	**Tocilizumab (*n* = 18)**	**Anakinra (*n* = 30)**	**Non-AIT (*n* = 20)**	***p*-value**
**Age, years, mean (s.e.m.)**	62.9 (3.5)	68.3 (2.5)	68.5 (3.9)	0.4266
**Male sex, *n* (%)**	12 (66.7)	19 (63.3)	12 (60.0)	0.9133
**Vaccinate, *n* (%)**	5 (27.8)	5 (16.7)	2 (10.0)	0.3507
Days to start the therapy, median [IQR]				
From symptom onset	7 [6-9]	10 [7-13]	-	0.1336
From hospital admission	0 [0-1]	2 [0-3]	-	0.5020
**Laboratory values at hospital admission, median [IQR]**				
WBC (x 10^3/µl)	5.42 [4.44-13.08]	8.84 [5.79-11.92]	6.45 [4.97-10.20]	0.4522
Lymphocytes (x 10^3/µl)	0.79 [0.38-0.94]	0.77 [0.60-0.91]	0.86 [0.65-1.20]	0.2812
CRP (mg/L)	99.18 [72.67-141.2]	144.90 [84.21-187.00]	40.43 [6.64-72.66]	**0.0019**
suPAR (ng/mL)	4.23 [3.07-5.65]	5.98 [3.82-7.61]	2.46 [1.80-5.35]	**0.0010**
sTREM-1 (pg/mL)	266.2 [207.9-371.4]	380.7 [238.4-539.1]	256.3 [178.9-355.5]	**0.0406**
HGF (pg/mL)	665.5 [266.3-1,773.0]	986.1 [411.2-2,276.0]	506.3 [227.1-1,088.0]	0.0961
MCP-1 (pg/mL)	218.7 [141.0-300.9]	410.2 [274.1-744.3]	339.3 [190.6-482.7]	0.1563
IL-1 (pg/mL)	3.45 [1.60-4.49]	3.45 [1.73-5.85]	1.64 [1.30-3.89]	0.3655
IL-6 (pg/mL)	12.77 [4.04-37.21]	24.65 [8.05-61.23]	5.01 [2.58-15.60]	0.0606
NLR	11.62 [4.49-15.46]	10.25 [5.11-13.57]	4.01 [2.57-13.77]	0.0982
PLR	307.9 [204.6-415.8]	260.5 [203.2-363.2]	194.1 [127.7-471.6]	0.4026
ESR (mm/h)	72.5 [43.8-120.0]	80.0 [51.3-105.8]	50.0 [21.0-92.0]	0.2201
Fibrinogen (g/L)	5.30 [4.23-5.82]	5.58 [4.923-6.623]	4.61 [3.3-5.82]	**0.0279**
Ferritin (µg/L)	1,003 [554-2,516]	1,292 [496-1,962]	318 [190-1,032]	**0.0037**
LDH (U/L)	390.5 [311.3-555.8]	395.0 [302.0-594.5]	226.0 [179.0-325.0]	**0.0001**
**Comorbidities, *n* (%)**				
Diseases of the blood involving the immune mechanism (D)	8 (44.4)	3 (10.0)	6 (30.0)	**0.0236**
Endocrine and metabolic disorders (E)	3 (16.7)	3 (10.0)	4 (20.0)	0.5970
Diseases of the circulatory system (I)	11(61.1)	14 (46.7)	10 (50.0)	0.6175
Diseases of liver and gallbladder (K)	2 (11.1)	1 (3.3)	1(5.0)	0.5302
**Co-administered antiviral/anti-inflammatory medications, *n* (%)**				
Remdesivir	10 (55.6)	8 (26.7)	2 (10.0)	0.2314
Dexamethasone	18 (100.0)	30 (100.0)	12 (60.0)	< 0.0001
Aspirin	3 (16.7)	1 (3.3)	0 (0.0)	0.6184
Diclofenac	4 (22.2)	4 (13.3)	3 (15.0)	0.5576
Metamizole	16 (88.9)	27 (90.0)	14 (70.0)	0.6708
Acetaminophen	13 (72.2)	24 (80.0)	11 (55.0)	0.4618

WBC, white blood cells; CRP, C-reactive protein; suPAR, soluble urokinase plasminogen activator receptor; sTREM-1, soluble triggering receptor expressed on myeloid cells-1; HGF, hepatocyte growth factor; MCP-1, monocyte chemoattractant protein-1; IL-1β, interleukin-1 beta; IL-6, interleukin-6; NLR, neutrophil-lymphocyte ratio; PLR, platelet-lymphocyte ratio; ESR, erythrocyte sedimentation rate; LDH, lactate dehydrogenase; *p*, statistical significance coefficient; *n*, number of cases; IQR, interquartile range.
